# Color-modulated olfactory testing using RAPPIT: An innovative tool for early detection of cognitive decline

**DOI:** 10.1177/13872877261449410

**Published:** 2026-05-20

**Authors:** Sally Arnhardt, Satnam Singh, Kristin Steinebach, Romy Fuchs, Tim Diener, Doris Gasser, Johannes Kornhuber, Jessica Freiherr

**Affiliations:** 1 Department of Psychiatry and Psychotherapy, 9171 Friedrich-Alexander-Universität Erlangen-Nürnberg, Erlangen, Germany; 2 Sensory Analytics and Technologies, Fraunhofer Institute for Process Engineering and Packaging IVV, Freising, Germany

**Keywords:** aging, Alzheimer's disease, cognition, cognitive screening, cognitive testing, mild cognitive impairment, Montreal Cognitive Assessment, neurodegeneration, olfaction, vision

## Abstract

**Background:**

Olfactory and visual processing are sensitive biomarkers for cognitive impairment; however, unimodal assessments may fail to capture early deficits in higher order cognitive integration. When sensory cues are mismatched, multisensory processing induces crossmodal conflict that requires inhibitory control, which is particularly vulnerable in early neurodegeneration.

**Objective:**

We developed RAPPIT (Rapid, massively APPlicable Identification Test), a color-modulated olfactory test to assess multisensory interference as an early marker of cognitive decline.

**Methods:**

RAPPIT includes 16 physically presented odors, with a digital application for task control and response recording. Answer options are displayed on color backgrounds derived from the MONEX-40 color profile, either congruent or incongruent with the presented odors. A total of 163 participants from the German population completed the assessment. Odor identification accuracy, effects of color congruence (ΔE00), associations with cognitive performance (Montreal Cognitive Assessment, MoCA), hedonic ratings, and depressive symptoms were analyzed.

**Results:**

Odor identification declined with age. Participants aged ≥ 60 years, a group at increased risk for neurodegenerative disorders including Alzheimer's disease, showed reduced performance under incongruent conditions. Performance exhibited a non-linear relationship with color difference (ΔE00), declining at mid-range values. Accuracy was significantly associated with MoCA scores. Hedonic ratings varied with color cues, while no associations were found with depressive symptoms.

**Conclusions:**

These findings demonstrate that differences between congruent and incongruent odor-color conditions capture cognitively relevant interference effects beyond unimodal olfactory or visual performance, supporting the utility of this approach for early detection of cognitive impairment in older adults, in clinical and home settings.

## Introduction

As individuals age, the risk of developing cognitive impairments increases, notably in the form of mild cognitive impairment (MCI), a condition affecting approximately 6.7% to 25.2% of adults.^1^ MCI is characterized by objectively measurable cognitive decline that does not yet interfere with daily functioning. However, it frequently represents a transitional stage toward dementia, with an annual progression rate of 10–15%,^2^ with Alzheimer's disease accounting for 60–80% of all dementia cases.^3^ Currently, around 55 million people worldwide live with dementia, and with an aging global population, 10 million new cases are expected to be diagnosed each year in the future.^4^ These numbers underscore the urgent societal and healthcare challenges associated with this growing epidemic. Early detection is critical, not only to delay or slow disease onset but also to improve the quality of life for both patients and caregivers while mitigating the associated economic burden. Extensive research demonstrates that pathological alterations, such as changes in molecular biomarkers including amyloid-β (Aβ_42_) and tau proteins, may be detectable up to 18 years before clinical diagnosis, highlighting their role as early drivers of disease pathology.^5^

Among emerging tools for early detection, the sense of smell stands out as a promising, non-invasive sensory biomarker. Olfactory decline often precedes both the onset of dementia and the appearance of measurable cognitive deficits. Even one smell test can indicate cognitive decline up to 15 years before symptoms appear.^6^ This is especially relevant in the context of Alzheimer's disease, which is characterized by early neuropathological changes in regions including the entorhinal cortex and the olfactory bulb.^7^ Consequently, olfactory dysfunction is considered one of the earliest and most sensitive indicators of neurodegeneration, with 85–90% of Alzheimer's disease patients exhibiting measurable deficits in olfactory function.^8^ Even in the subjective cognitive decline (SCD) stage, marked by self-perceived memory impairment without objective deficits, olfactory decline has been documented.^9,10^ This stage is associated with an elevated risk of progressing to MCI,^11^ which is itself frequently accompanied by pronounced olfactory impairments.^12,13^ Existing diagnostic tools (such as UPSIT^14^ and the identification subtest of the Sniffin’ Sticks^15^) primarily rely on picture- or word-supported odor identification. Other approaches, including the full Sniffin’ Sticks battery^16^ and the AROMHA Brain Health Test,^17^ also assess threshold and discrimination, but nevertheless remain limited to unimodal olfactory measures and overlook multisensory interactions.

Further, olfaction is not the only sensory modality linked to cognitive decline. Vision also represents a valuable potential marker for neurodegenerative processes, with impairments observed in both the early (MCI) and later stages of dementia. Visual dysfunctions have been documented in domains such as contrast sensitivity, pupillary response, retinal integrity, and color perception^7,18–23^. Olfactory processing, being largely subconscious, is strongly influenced by the dominance of visual perception in daily life. Colors, shapes, images, and labels are closely linked to odor processing and memory, often forming associative pairings. These crossmodal associations emerge in infancy^24^ and are increasingly shaped by language and cultural context throughout development.^25^ Research has shown that specific odors are consistently associated with certain colors.^26,27^ For instance, the smell of strawberries is commonly linked to shades of red or pink, but rarely to colors like turquoise, indicating that some odor–color pairings are perceived as congruent while others are not.^28^

Not only the processing of individual sensory modalities but also their integration across modalities is affected by neurodegenerative processes. For instance, with accumulating sensory impairment, brain regions such as the orbitofrontal gyrus and entorhinal cortex show reduced volumes.^29^ Impairments in multisensory integration have been significantly associated with cognitive decline.^30^ A previous study demonstrated that cognitively impaired individuals exhibit weaker multisensory integration for weak incongruent audiovisual stimuli, reflected in a reduced visual bias.^31^

Building on these findings, we hypothesize that a similar pattern will emerge in a color-modulated olfactory identification task. Given the strong crossmodal links between olfaction and vision and the fact that both sensory domains exhibit early signs of neurodegeneration, we developed a novel color-modulated odor identification test, called RAPPIT (Rapid, Massively APPlicable Identification Test). This test is designed to provide a more sensitive and valid tool for differentiating degrees of cognitive impairment by incorporating congruent and incongruent color cues. We expect congruent color backgrounds to facilitate odor identification, whereas incongruent ones may interfere with processing, particularly in individuals with cognitive impairment, by disrupting multisensory integrity. The colors were selected from the MONEX-40 color profile, developed in a previous experiment conducted in our lab.^26^ To evaluate this approach, we conducted a cross-sectional study in a diverse German-speaking sample (n = 163) across the adult lifespan, using the Montreal Cognitive Assessment (MoCA) to capture early cognitive trends.

## Methods

To develop a mobile and user-friendly screening tool for future public use, we implemented a hygienic and self-administered odor presentation format. Small brown glass jars were selected as recyclable, light-protective containers, each sealed with an airtight ring to ensure odor stability. To allow safe handling and consistent odor release, the liquid odorants from the MONEX-40 Sniffin’ Sticks were reformulated into a solid medium by emulsifying them in Hard Fat W35 (IOI Oleo GmbH, Hamburg, Germany), a non-toxic, nearly odorless, and stable base with excellent emulsifying properties. A pilot study was conducted prior to the main experiment to ensure comparable odor intensity between the Sniffin’ Sticks and the airtight jar format for mobile testing. Odor intensity was successfully maintained at least at the level of the original format. The experimental procedure was digitally implemented via a dedicated mobile application, which guided participants through each step of the test, including instructions for odor presentation, response recording, and timing control.

For the present study, all testing was conducted under supervision, with experimenters bringing the odor samples to the participants and overseeing the procedure on site. While the present study focused on the development and validation of the prototype, the final version is intended to enable large-scale, home-based assessment, with light-protected odor samples shipped directly to participants. The app's user interface was designed with a strong emphasis on usability and accessibility, specifically tailored to the needs of older participants, who represent a key target group for olfactory screening (for more details, see Supplemental Material 1).

### Participants

To evaluate the newly developed color-modulated odor identification test, 171 participants were recruited between September 2024 and March 2025 via digital platforms, print media, and telephone outreach (e.g., senior centers). The recruitment strategy was designed to reflect a broad population sample. Participants had to be German speakers, not be pregnant or breastfeeding, and free from acute infectious diseases, color blindness, synesthesia, and recent antibiotic use. Individuals with a current or past history of chemo-/radiotherapy in the head or neck region, multiple chemical sensitivity, or zinc deficiency were excluded. A minimum score of four out of five on a validated odor identification screening test^32^ was required. After excluding one participant due to technical failure, six who failed the screening criterion, and one extreme outlier, data from 163 participants (mean age = 47 years, SD = 22.40 years; age range: 19–90 years) were included in the final analysis (Table 1). Participants received €15 compensation. For analysis, all participants (n = 163) were considered as a whole group and additionally divided into two age groups: ≥ 60 years (n = 66; higher risk of cognitive decline) and < 60 years (n = 97). The study was approved by the Ethics Committee of the Medical Faculty at Friedrich-Alexander-Universität Erlangen-Nürnberg (ethics protocol 24–271-B). All participants provided written informed consent, and procedures followed the Declaration of Helsinki. Detailed demographic profiles are provided in Supplemental Material 2.

**Table 1.** Sample characteristics of participants (n = 163).

	Total	Congruent version	Incongruent version
**n**	163	81	82
Male	59	30	29
Female	104	51	53
Test in Lab room	129	64	65
Test in Real-life situation	34	17	17
**Age**	47 [22.40]	46.51 [21.76]	47.49 [23.13]
**Education**	16.16 [3.08]	16.16 [2.97]	16.12 [3.20]
**MoCA**	26.40 [2.86]	26.46 [2.91]	26.34 [2.83]
**BDI-II**	5.02 [5.27]	4.78 [4.80]	5.26 [5.71]
**SCD**	23.94 [19.01]	25.09 [19.62]	22.81 [18.45]
**Test Score**	12.64 [1.92]	12.77 [1.95]	12.52 [1.91]

The table presents the total number of participants and their distribution across the two test groups (“congruent” and “incongruent”), as well as the number of participants tested in a standardized laboratory setting versus a real-life environment. For each subgroup, the following variables are reported as mean values with standard deviations [SD]: age, years of education, MoCA (Montreal Cognitive Assessment), BDI-II (Beck Depression Inventory-II), SCD (subjective cognitive decline), and the overall odor-color test score.

### Experimental procedure

**Figure 1.**
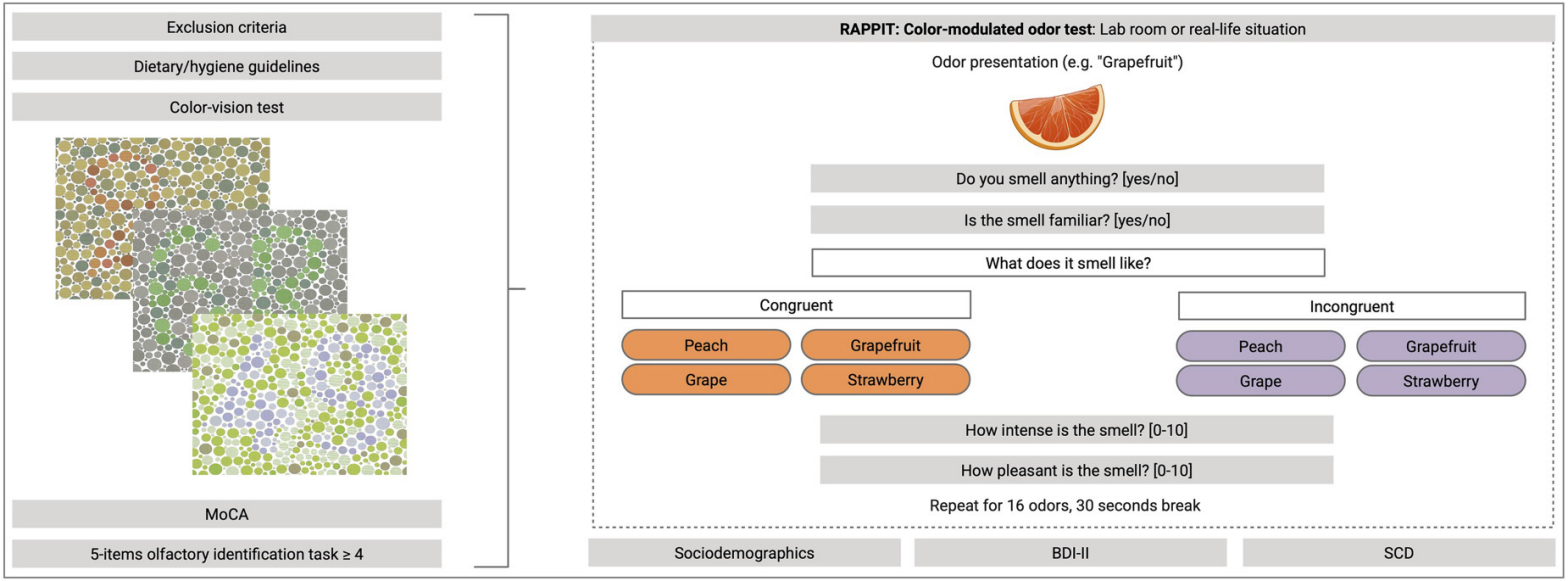
Experimental study design. Participants who met all inclusion criteria completed the odor-color test involving 16 randomized odor presentations with 30-s intervals. They were assigned to either a congruent or incongruent color version, rated odor familiarity, intensity, and pleasantness using visual analogue scales (VAS), and completed sociodemographic, BDI-II, and SCD questionnaires. Created with BioRender.com by Sally Arnhardt.

Participants completed the odor-color test either in a controlled laboratory environment or in real-life settings (e.g., care facilities, homes), always under supervision. This dual-setting design was chosen to evaluate the test's applicability as a future mobile home-use tool. As established in the pilot study, participants followed standardized dietary and hygiene guidelines to minimize potential confounding factors (see Supplemental Material 1). Appointments were distributed across the day to reduce time-of-day effects. Before testing, participants were screened for color blindness using a digital version of color charts.^33^ Only those who correctly identified red-green and blue-yellow stimuli continued. Visual aids with optical filters were not permitted due to potential color distortion. Cognitive functioning was assessed through screening using the standardized MoCA.^34^ The odor-color test was conducted via a digital application on standardized tablets. Participants were randomly assigned to a congruent (odor-color match) or incongruent test group (odor-color mismatch),^26^ with subversion variations for the latter (Figure 1). Each trial involved: (1) odor detection and familiarity questions, (2) multiple-choice color-coded response selection, (3) intensity and pleasantness ratings via VAS (0–10). Odors were presented in randomized order with 30-s breaks. Participants could re-smell each odor during the trial. The full odor test lasted 15–20 min. After the test, participants completed a digital questionnaire on socio-demographics, depression (BDI-II),^35^ and SCD.^36^ The SCD inventory was translated and adapted for this study following standard translation/back-translation protocols. Mobile sessions followed the same protocol as lab testing, with efforts to minimize distractions (quiet rooms, lighting control). Each full session lasted ∼60 min. Technical details regarding experimental procedures are provided in Supplemental Material 3.

### Statistical analyses

#### Group comparisons

To compare outcomes between the congruent and the incongruent test groups, statistical analyses were conducted for overall test scores as well as average ratings of intensity, pleasantness, and familiarity. All analyses were performed for the full sample (all ages), younger participants (<60 years), and older participants (≥60 years), based on the increased risk of developing cognitive impairments around the age of 60 years.^1,37^ Normality was assessed using the Shapiro–Wilk test, and homogeneity of variances via Levene's test. Depending on these assumptions, either parametric (independent samples t-tests, Welch's t-tests) or non-parametric (Mann-Whitney U tests) tests were used. Binary variables were analyzed using Chi-square tests (with Yates’ correction) or Fisher's exact tests when expected cell counts were low (<5). To ensure the robustness of the findings, two different correction methods for multiple comparisons were applied: a conservative approach (Bonferroni correction) and a less conservative one (False Discovery Rate [FDR] according to Benjamini–Hochberg^38^). This allows for a comparison of results across methods and helps assess the stability of the conclusions regardless of the correction applied. All visualizations were created using Biorender.com; statistical analyses were conducted using JASP (v0.19.2) and Python (v3.12.1, PyCharm 2023.3.2 Community Edition).

#### Regression analyses

To explore associations between test scores and ratings (intensity, pleasantness, familiarity) with demographic and cognitive-affective variables, separate analyses were conducted for the congruent and the incongruent test version. Subject-wise mean values were computed for the rating variables before analysis. The examined associations included: Test score versus Age, SCD, MoCA, BDI-II; MoCA versus Age; Mean Intensity, Pleasantness, Familiarity versus Age and MoCA; Mean Intensity versus Mean Pleasantness. For each pair of variables, normality was assessed using the Shapiro–Wilk test. Depending on distribution, either Pearson or Spearman correlation coefficients were calculated. Subsequently, linear regression analyses were performed, and model assumptions were checked via residual diagnostics (Shapiro–Wilk test, Q–Q plots). In cases of non-normal residuals or heteroscedasticity (Breusch–Pagan test, p ≤ 0.05), models were refitted using heteroscedasticity-consistent standard errors.^39^ To compare correlation strengths between the two versions, z-tests for independent correlations were conducted. All statistical analyses and visualizations were carried out in Python (v3.12.1) using pandas, scipy, statsmodels, seaborn, and matplotlib.

#### Generalized linear mixed model (GLMM)

To examine the influence of color difference (ΔECIE2000) and cognitive ability (MoCA) on response accuracy (isCorrect), a generalized linear mixed model (GLMM) with binomial distribution and logit link was fitted using R (v4.4.3) in RStudio. Data from the incongruent version (which included ΔECIE2000 values calculated based on the congruent version) were used. The ΔECIE2000 (ΔE00) value is a numerical measure that represents how different two colors appear to the human eye, based on their coordinates in the CIELAB color space.^40^ It quantifies color differences by considering hue, saturation, and lightness. Lower values indicate smaller perceptual differences, with differences below 1 generally considered imperceptible and values between 1 and 2 detectable only by experienced observers. Differences of 2–5 are typically noticeable at a glance, whereas values greater than 5 represent clearly distinct colors. This metric therefore allows for a robust and perceptually meaningful comparison of color changes across samples or experimental conditions. Visual exploration of the relationship between predictors and response accuracy was performed using the ggplot2 package. Smoothed trend lines were added via geom_smooth with a LOESS method, and raw response distributions were visualized using geom_count to reflect data density for binary outcomes. The model included a random intercept for each participant to account for repeated measures. Color difference was modeled using cubic B-splines (df = 3) to allow for non-linear effects, while MoCA was included as a linear predictor. The model was estimated using the lme4 package with the bobyqa optimizer. Variance inflation factors (VIFs) were computed using the performance package to assess multicollinearity, and residual diagnostics were performed using the DHARMa package. Model performance was assessed via ROC curve analysis and AUC calculation using the pROC package. The final model included 1312 observations from 82 participants.

## Results

**Figure 2.**
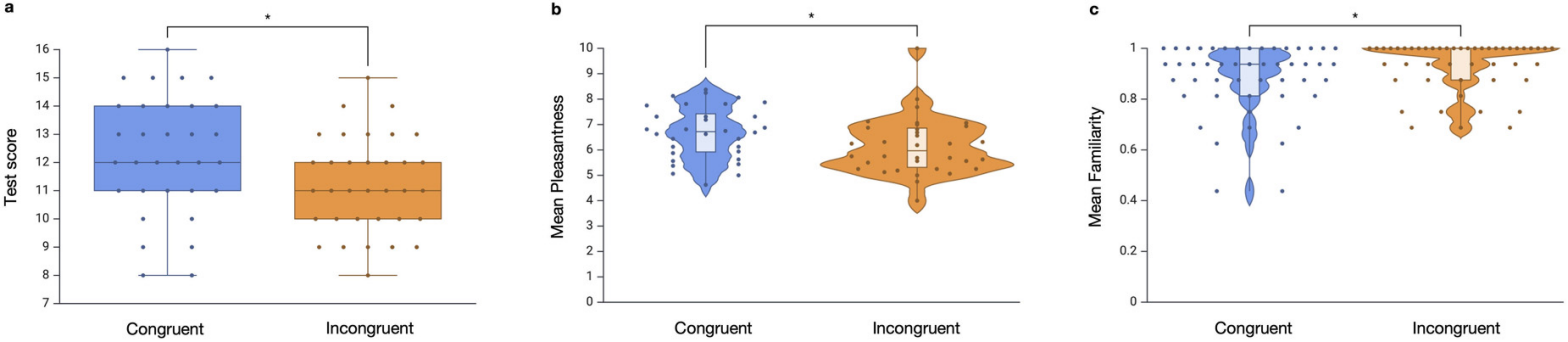
Olfactory performance during congruent versus incongruent color display. Panels depict group comparisons: (a) Test score (congruent versus incongruent, ≥ 60 years), (b) Mean pleasantness (congruent versus incongruent, ≥ 60 years), (c) Mean familiarity (congruent versus incongruent, < 60 years). Created with BioRender.com by Sally Arnhardt.

We did not find a significant difference in olfactory identification test scores between participants involved in the congruent and incongruent test versions (congruent: M = 12.77, SD = 1.94; incongruent: M = 12.52, SD = 1.91, U = 3057.00, p = 0.38). Focusing on participants under 60 years of age, scores did not differ between test versions (congruent: M = 13.10, SD = 1.77; incongruent: M = 13.44, SD = 1.49, U = 1069.50, p = 0.44). In contrast, focusing on participants above 60 years, they performed significantly better in the congruent test (M = 12.25, SD = 2.11) compared to the incongruent group (M = 11.24, SD = 1.69), t(64) = 2.16, p = 0.034, with a mean difference of −1.01 (95% CI [0.08, 1.95]; Cohen's d = −0.53) (Figure 2). No significant differences between the congruent and incongruent subgroups in terms of age, education, MoCA scores, or medical characteristics, and no confounding health variables were identified in the 60 + age group (Supplemental Material 4). Intensity ratings did not differ significantly between test versions in any age group. Pleasantness ratings were significantly lower in the incongruent version among older participants (congruent: M = 6.69, SD = 1.04; incongruent: M = 6.15, SD = 1.12, U = 375.50, p = 0.03), but not in younger participants or the full sample. For familiarity, a significant difference was found only in the younger group, where odors were rated as more familiar by the incongruent group (congruent: M = 0.88, SD = 0.14; incongruent: M = 0.93, SD = 0.10, U = 869.50, p = 0.02), with no differences in older participants or the full sample.

We additionally examined potential sex differences in test performance for both the congruent and incongruent versions within our group of interest (participants aged ≥ 60 years). Due to non-normal data distribution, a Mann–Whitney U test was used for the congruent group, revealing a significant difference between sexes (men: M = 10.80, SD = 1.69; women: M = 12.91, SD = 1.97, U = 44.00, p = 0.007), with women showing higher test scores than men. For the incongruent group, which met the assumption of normality, a Student's t-test was performed and showed no significant difference between sexes (t(32) = −0.43, p = 0.674).

To examine the ecological validity and robustness of the test across different environments, performance was compared between laboratory and home-based assessments in participants aged 60 years and older. No significant differences in test performance were found between settings of the task (t(64) = –0.68, p = 0.50).

**Figure 3.**
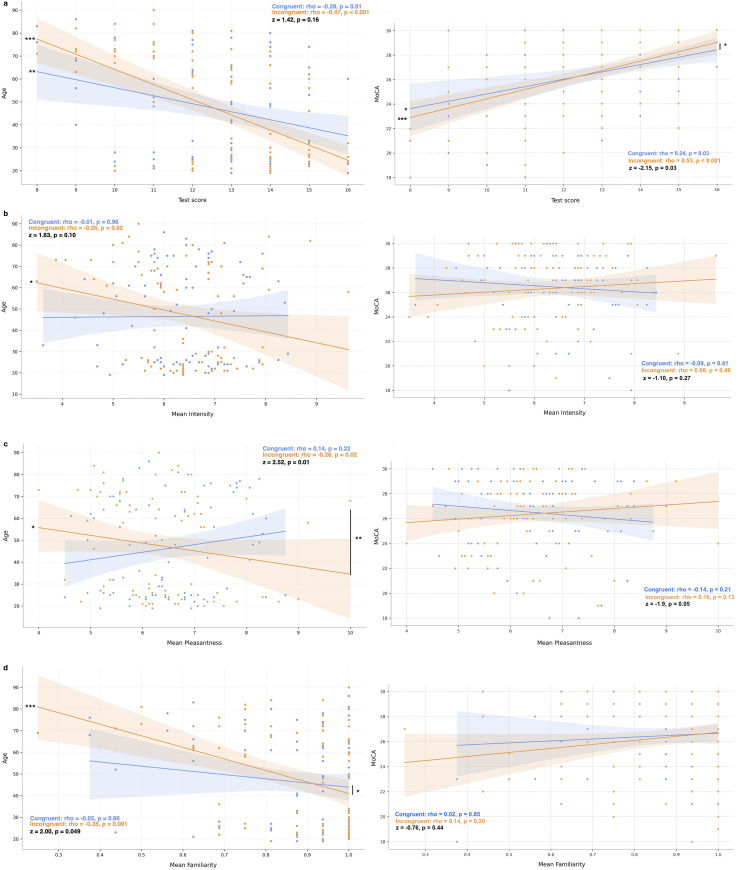
Associations between test performance and cognitive/demographic variables. Panels show scatterplots with 95% shaded confidence intervals; asterisks indicate significant correlation coefficients. (a) Test score versus Age/MoCA, (b) Mean intensity versus Age/MoCA, (c) Mean pleasantness versus Age/MoCA, (d) Mean familiarity versus Age/MoCA. Significant effects are marked with asterisks.

Correlational analyses were conducted to examine associations between the test and cognitive as well as demographic variables. Additionally, regression analyses were performed to assess predictive power for the congruent and incongruent versions. Test scores showed significant negative correlations with age (congruent: rho = −0.28, p = 0.01; incongruent: rho = −0.47, p < 0.001). Regression models confirmed these effects, with stronger predictive value in the incongruent test group (R^2^ = 0.30, p < 0.001 versus R^2^ = 0.10, p = 0.004), though the difference between versions was not statistically significant (Figure 3). SCD was also negatively associated with test performance (rho = −0.24, p = 0.03 in both versions), and regression analyses yielded modest but significant effects (congruent: R^2^ = 0.05, p = 0.045; incongruent: R^2^ = 0.05, p = 0.03). These findings suggest that the test is sensitive to early, self-perceived cognitive changes, although performance was not modulated by color congruency. In contrast, stronger effects were observed for objective cognitive functioning. Test scores showed a positive correlation with MoCA scores (congruent: rho = 0.24, p = 0.03; incongruent: rho = 0.53, p < 0.001), with a significantly stronger association in the incongruent group (z = −2.15, p = 0.03). Regression confirmed this pattern (R^2^ = 0.15, p = 0.002 versus R^2^ = 0.26, p < 0.001), suggesting that the incongruent version is particularly sensitive to cognitive decline under perceptual conflict. As expected, MoCA scores declined significantly with age (congruent: rho = −0.56, p < 0.001; incongruent: rho = −0.57, p < 0.001), but this relationship did not differ between congruency groups (z = 0.10, p = 0.92), indicating that the association between test score and cognition is not merely age-driven. No significant associations were found between test performance and depressive symptoms (BDI-II), suggesting that the test is relatively unaffected by affective state and may provide more specific insight into neurocognitive functioning.

Intensity ratings were positively associated with pleasantness across both groups (congruent: r = 0.40, p < 0.001; incongruent: rho = 0.38, p < 0.001), with no difference between groups (z = 0.16, p = 0.87). Regression confirmed a stable association (congruent: R^2^ = 0.16, p < 0.001; incongruent: R^2^ = 0.17, p < 0.001), indicating that perceived odor strength consistently relates to hedonic evaluation, independent of color congruency. In the incongruent version only, intensity declined with age (rho = −0.26, p = 0.02), while no such effect was observed in the congruent version (rho = −0.01, p = 0.96). However, intensity ratings did not correlate with MoCA scores in either version, suggesting that intensity reflects age-related perceptual decline but is not sensitive to cognitive performance. Pleasantness ratings showed a negative correlation with age in the incongruent version only (rho = −0.26, p = 0.02), while no effect was found in the congruent version (rho = 0.14, p = 0.22). Although regression results were not statistically significant in either case, the difference between versions was (z = 2.52, p = 0.01), suggesting that age-related decline in pleasantness perception is modulated by perceptual congruency. Similarly, no significant associations were found between pleasantness and MoCA scores within either version. Familiarity ratings declined significantly with age in the incongruent version (rho = −0.35, p = 0.001), but not in the congruent version (rho = −0.05, p = 0.66), with a significant difference between conditions (z = 2.00, p = 0.049). Regression supported this pattern, indicating a moderate age-related decline under incongruent conditions (R^2^ = 0.14, p < 0.001). No associations with MoCA were observed, suggesting that familiarity reflects age-related perceptual changes rather than cognitive performance. Supplementary analyses, including detailed predictive regression results, can be found in Supplemental Material 5.

**Figure 4.**
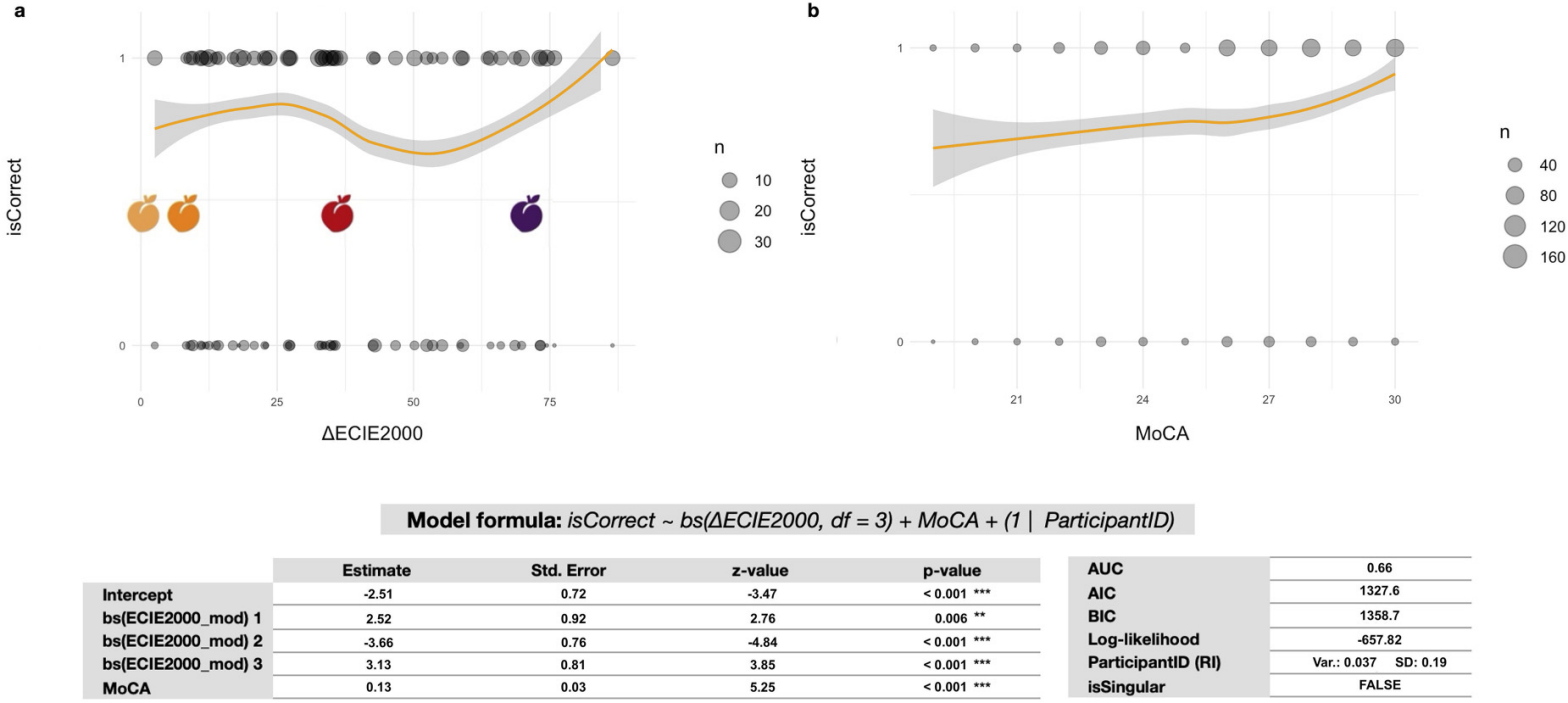
Associations between a) Color distance and response accuracy and b) MoCA and response accuracy. (a) Relationship between ΔECIE2000 and correct responses. As an example, odor 1 (peach) as a congruent reference color (ΔECIE2000 = 0) and three incongruent subversions with increasing color differences to the reference, shown from left to right: ΔE = 8.31, 35.64, and 69.81. (b) Relationship between MoCA and correct responses. Circle size reflects observation count; LOESS curves with 95% confidence intervals illustrate trends. The binary outcome (isCorrect) is plotted on the y-axis. Significant effects were found for ΔECIE2000 and MoCA scores. Model diagnostics (DHARMa) indicate no violations of assumptions.

To examine how perceptual color differences (ΔECIE2000) and cognitive ability (MoCA) affect response accuracy, a mixed-effects logistic regression was fitted to 1312 observations from the incongruent test version (n = 82). Random intercepts accounted for individual differences. Visual inspection suggested a non-linear relationship between ΔECIE2000 and accuracy, with lowest accuracy at medium color differences. MoCA showed a positive linear association with correct responses. Based on these patterns, ΔECIE2000 was modeled using cubic B-splines and MoCA as a linear predictor. The full model significantly outperformed a null model (χ^2^(4) = 54.01, p < 0.001). All spline terms were significant (Figure 4), confirming the non-linear perceptual effect, and MoCA showed a robust positive effect (β = 0.13, z = 5.25, p < 0.001), indicating that higher cognitive ability predicts greater response accuracy. Model diagnostics confirmed good fit and no violations of assumptions. These results suggest that response accuracy is shaped by both a non-linear sensory factor (color difference) and a linear cognitive factor (MoCA), highlighting the test's potential to capture subtle perceptual-cognitive interactions. Additional age-stratified analyses (<60 versus ≥60 years) revealed similar but less stable patterns with lower predictive accuracy. A detailed description of these analyses, along with model assumption checks, can be found in Supplemental Material 6.

## Discussion

This study presents the development and evaluation of a novel color-modulated odor identification test, called RAPPIT, that combines odor perception with color congruence, designed for the rapid and user-friendly early detection of neurodegeneration. The test, tailored to target age- and cognition-specific markers, is based on a 16-item odor identification task in which response options are displayed against backgrounds of either congruent or incongruent colors, derived from the MONEX-40 color profile.^26^ We evaluated the test across a broad age range within the German population, including individuals with varying levels of cognitive function, demonstrating its potential for future validation in clinically diagnosed populations such as those with MCI or Alzheimer's disease.

Unlike olfactory threshold tests, which primarily reflect peripheral factors such as nasal anatomy and the number of receptors in the epithelium, odor identification performance specifically targets higher-order central processes related to odor memory and cognition, involving brain regions like the piriform cortex, orbitofrontal cortex and hippocampus.^41^ Discrimination tasks were omitted because they mainly require short-term maintenance and perceptual comparison of odor representations, while odor-color association effects are typically studied as crossmodal influences on odor processing and identification, which aligns with the integrative and interference-based mechanism targeted here. By focusing solely on identification, we avoid the need for multiple test formats, which could be confusing or overwhelming for older adults, thereby ensuring a simpler and more user-friendly assessment fitted to this population.

In a pilot study, we developed a recyclable, hygienic, and cost-effective jar-based odor presentation method and a dedicated app enabling digital testing in both laboratory and home settings. The airtight jars created an immediately perceivable scent depot, and odor intensity was rated at least as strong as odors presented in a Sniffin’ Sticks format. The study demonstrated the method's feasibility as a self-administered home-testing alternative to conventional approaches.

Our analysis showed significant correlations between test score and age across both congruent and incongruent versions, aligning with numerous studies reporting age-related decline in olfactory performance.^42,43^ Since cognitive decline risk increases with age and is closely associated with reduced olfactory abilities,^44^ we furthermore examined the relationship between our odor-color test and early cognitive impairment, especially in participants aged 60 years and older who are at high risk of developing neurodegenerative processes.^1,37^

SCD is considered one of the earliest detectable stages before MCI, a prodromal phase of dementia. Recent findings suggest that olfactory deficits may occur even when objective cognitive impairments are not yet measurable.^9,10^ Our results also indicated that poorer odor identification performance was significantly associated with higher SCD scores. Notably, there were no significant differences between congruent and incongruent test versions in this context, suggesting that color congruence may play only a minor role at this very early, purely subjective stage of cognitive decline. Importantly, instead of brief screening questions, we used the validated and comprehensive McCusker Subjective Cognitive Impairment Inventory (McSCI)^36^ to provide a more detailed and standardized assessment of SCD.

Following this, we examined how our test correlates with the MoCA, a sensitive tool for detecting objective cognitive impairments. The MoCA is frequently utilized as a neuropsychological screening tool for detecting MCI, a recognized high-risk stage preceding dementia.^34^ We selected this test owing to its greater sensitivity in detecting cognitive impairments in adults aged 60 years and above, compared to the Mini-Mental State Examination.^45,46^ Our findings exhibit a significant correlation between the odor-color test scores and MoCA: lower cognitive functioning was associated with lower odor identification performance. This supports previous research indicating that olfactory identification declines in the presence of cognitive impairment^47–49^. Of particular interest, the correlation was significantly stronger in the incongruent version than in the congruent version. However, a regression analysis showed that the MoCA score correlated equally with both test versions in relation to age. This suggests that color congruence plays a significant role when cognitive impairment, rather than age alone, is present, indicating that this tool may be useful for more sensitive early-stage diagnostic screening.

Group comparisons also revealed that participants aged 60 + years had significantly more difficulty identifying odors when presented with incongruent background colors. We controlled for sex, age, education, MoCA scores, test environment, smoking status, and medical/medication profiles, indicating that performance differences were indeed driven by the color congruence factor. These findings align with prior studies showing that aging and cognitive decline affect visual processing as well.^21,50^ In addition to the main analyses, we examined potential sex differences in test performance for both versions within our group of interest (60 + years). The better performance of women in the congruent version may be related to generally superior olfactory sensitivity reported in the literature, which could provide an advantage in tasks relying on odor identification and discrimination.^51^ In contrast, the absence of this advantage, and the relative drop in performance, in the more cognitively demanding incongruent version might reflect a higher vulnerability to cognitive load in females. This pattern of differences could tentatively be linked to the higher risk of Alzheimer's disease in women,^52^ where early compensatory advantages in sensory or cognitive domains may diminish under increased task complexity. Furthermore, we found that participants aged 60 years and older performed comparably in the laboratory and home-based settings. This consistency indicates that the test yields robust results across different environments, supporting its ecological validity. Consequently, the color-modulated odor identification test appears well-suited for mobile and home-based applications, offering a practical approach for large-scale screening and early detection of cognitive impairment in older participants.

Generally, the brain is more efficient at resolving information conflicts within a single sensory modality than between different sensory modalities—a pattern that has also been demonstrated for other modality combinations, such as auditory–olfactory pairings.^53^ Moreover, not only olfactory and visual processing appear to be impaired in the context of cognitive decline; there is also evidence that multisensory integration processes may become pathologically altered. Several studies have shown that multiple sensory impairments are associated with an increased risk of dementia.^29^ The Baltimore Longitudinal Study of Aging found that each additional sensory impairment was associated with reduced volume in the orbitofrontal gyrus and the entorhinal cortex.^54^

Our behavioral results show a U-shaped performance pattern, with the lowest accuracy at intermediate levels of odor–color congruence. This pattern complements Noppeney's (2021) account of an inverted U-shaped function in multisensory integration, where moderate crossmodal disparity elicits the strongest integration processes.^53^ Behaviorally, this may manifest as increased perceptual conflict and reduced task efficiency, reflecting the brain's effort to resolve competing sensory evidence.

Festa et al. (2017) reported a similar pattern in the audiovisual domain, showing that individuals with MCI exhibited reduced visual influence under weak audiovisual incongruence compared to healthy controls, while performance under strongly incongruent or fully congruent conditions remained comparable between groups.^31^ Healthy individuals display a dynamic adjustment to varying levels of sensory conflict, whereas cognitively impaired individuals show a reduction in bottom-up integration, which they compensate for through top-down processing. This difference was also reflected in our results: cognitively impaired participants were particularly challenged by incongruent color–odor pairings, suggesting that they were less able to suppress misleading visual cues and therefore integrated conflicting information inappropriately. In contrast, healthy young adults showed greater flexibility, displaying a trend for more accurate odor identification under incongruent conditions—consistent with an adaptive arbitration between integration and segregation.^55^ A similar trend emerged for familiarity ratings, with odors in the incongruent condition being rated as significantly more familiar. This may reflect younger adults’ greater cognitive flexibility and control, enabling them to overcome the crossmodal interference induced by incongruent color–odor pairings, an effect reminiscent of the Stroop phenomenon.^56^ In contrast, older adults and cognitively impaired participants, who show reduced executive control and sensory precision,^57^ may be less able to resolve such conflicts. These age- and cognition-related performance differences suggest that our task can effectively differentiate between age groups and cognitive states, further supporting its potential as a sensitive tool for detecting early cognitive decline.

Hedonic ratings further differentiated cognitive profiles. Pleasantness emerged as the most cognitively sensitive dimension, with significantly lower ratings in the incongruent condition among participants aged 60 + years, indicating that disrupted color–odor congruence impacts emotional appraisal. This aligns with findings that odor pleasantness is a key component of olfactory perception^58^ and serves as a sensitive marker of cognitive status in aging populations.^59^ Familiarity and intensity showed milder effects but contributed to the overall test sensitivity. Regression analyses confirmed that scores from the newly developed test significantly predicted participants’ age and cognitive performance, both at the test score level and, to some extent, at the hedonic level.

Unlike classical odor identification tests that mainly reflect peripheral olfactory abilities, RAPPIT additionally engages cognitive mechanisms by requiring participants to integrate visual and olfactory information and resolve crossmodal conflicts. This design recruits higher-order processes such as semantic memory retrieval, attention, and executive control, supported by orbitofrontal, piriform and entorhinal, and prefrontal regions**—**areas particularly vulnerable in early neurodegeneration. The stronger association between RAPPIT performance and MoCA scores in the incongruent condition further supports that the test captures not only olfactory but also cognitive components, particularly those related to conflict resolution and multisensory integration. Although olfactory loss has been associated with depression,^60^ no link with BDI-II scores emerged in our data, suggesting specificity of the test for neurocognitive decline.

Regarding the limitations of our study, it is important to note that participants were not clinically diagnosed but rather drawn from a general population sample, which may limit the precision of cognitive status classification. MoCA-based trends were used to infer cognitive functioning. Future studies should aim to validate the test in clinically diagnosed MCI and dementia cohorts. Moreover, incorporating digital assessment tools like RAPPIT into these diagnostic settings may provide a scalable and accessible complement to biomarker- and genotype-based approaches. We intentionally used a population-based sample to assess whether the odor-color congruence concept works across the general population. Focusing on a representative community sample ensured that the test's performance could be evaluated under real-world conditions before its application in clinical settings. This design allowed us to establish normative data and identify preliminary trends among individuals at higher risk for cognitive decline. Tests conducted in real-life environments did not always provide standardized conditions, unlike those in lab settings. However, this was intentional, as the test is designed to be robust for at-home, self-administered use. Regarding inclusion criteria, we used a short version of a color vision test (three color charts). Though longer versions exist, time constraints prevented their use. Participants were also asked about synesthesia but were not formally tested; therefore, we cannot be entirely certain that all participants were free of synesthetic experiences. All participants had to pass both the 5-items olfactory identification test and the color vision test to be included. It is important to note that some individuals with advanced cognitive decline, due to severe impairments, may not be able to complete these tasks, which is why their inclusion in future studies could be considered as an option. Since our focus was on early detection in a general population and we wanted to exclude other olfactory impairments (e.g., post-COVID), we accepted this strict exclusion criterion and additionally controlled for potential cases who might exhibit reduced olfactory performance. Furthermore, within our group of interest, we tested for potential significant differences related to known medical conditions and medications to control for their possible influence on smell performance; however, no significant differences were found. It is also worth mentioning that the study on the color profile used here collected data for label-color associations without any age restrictions, but the odor-color associations were conducted with healthy young participants up to 45 years old.^26^ These associations may also vary depending on age, which could potentially influence test performance. In the present study, odor identification was assessed before participants rated the pleasantness and intensity of the odor. This sequence was chosen to capture participants’ initial and most spontaneous identification responses, as odor identification was the primary outcome measure of the test. However, it must be acknowledged that providing an odor label prior to rating pleasantness could influence hedonic evaluations, as previous research suggests that semantic information can bias affective odor judgments.^61^ Future versions of the test may therefore consider reversing the order of these tasks or implementing control conditions to minimize potential label-related bias in pleasantness ratings. While the MoCA is well suited for detecting MCI and early neurodegenerative changes, it may show ceiling effects in younger participants. Future studies could therefore complement this screening tool with more demanding cognitive measures to provide additional insight into the sensitivity and construct validity of RAPPIT. Although we thoroughly tested the new odor presentation in a pilot study and demonstrated that its intensity is at least as strong as in the previous study, some odors were perceived as more intense compared to the Sniffin’ Sticks. Familiarity and pleasantness were comparable overall but showed varying hedonic values depending on the specific odor. This variability may lead to differences in how color associations are represented in terms of saturation and brightness. Since a new presentation method cannot achieve 100% consistency, we assume that a standardized reduction of all concentrations by a factor of 10 should provide a comparable representation. Also worth mentioning is that the BDI-II is officially validated only up to the age of 80 years. Nonetheless, some participants over 80 years of age were included in the BDI-II regression analysis. It would also be of interest that future studies incorporate a time-stamping feature in the test app to analyze age-related interference effects in more detail.

## Conclusion

This study introduces a simple, color-modulated odor identification test for rapid and early detection of age- and cognition-related decline. Designed for older participants, it holds promise for future clinical validation in MCI and Alzheimer's disease populations.

## Supplemental Material

sj-docx-1-alz-10.1177_13872877261449410 - Supplemental material for Color-modulated olfactory testing using RAPPIT: An innovative tool for early detection of cognitive decline Supplemental material, sj-docx-1-alz-10.1177_13872877261449410 for Color-modulated olfactory testing using RAPPIT: An innovative tool for early detection of cognitive decline by Sally Arnhardt, Satnam Singh, Kristin Steinebach, Romy Fuchs, Tim Diener, Doris Gasser, Johannes Kornhuber and Jessica Freiherr in Journal of Alzheimer's Disease
