# Supplementary material for: Color-modulated olfactory testing using RAPPIT: An innovative tool for early detection of cognitive decline
Source: J Alzheimers Dis. 2026 May 20;112(1):148–60. doi: 10.1177/13872877261449410 (PMC13291397; doi:10.1177/13872877261449410)
Supplement: sj-docx-1-alz-10.1177_13872877261449410 - Supplemental material for Color-modulated olfactory testing using RAPPIT: An innovative tool for early detection of cognitive decline [file sj-docx-1-alz-10.1177_13872877261449410.docx]

**Supplemental Material**

**Color-modulated olfactory testing using RAPPIT: An innovative tool for early detection of cognitive decline**

**Supplemental Material 1: Pilot study**

*Participants*

As a preparatory step toward developing a future mobile do-it-yourself home test kit, this study aimed to design a novel odor delivery system that is more cost-effective, hygienic, user-friendly, and recyclable than conventional Sniffin’ Sticks, which were used in the foundational MONEX-40 color profile study^1^. For this purpose, a total of 30 native German speakers (15 men and 15 women) were recruited between May and September 2024 through various digital platforms (LinkedIn, Instagram, WhatsApp) as well as print media. Participants had to be native German speakers or use German as their primary language in daily life. This criterion was applied because the new test is based on the MONEX-40 color profile, which was likewise developed within the German population. Participants enrolled in the pilot study had a mean age of 27.03 years (SD = 5.30; range: 20–41 years). Inclusion criteria required participants to be between 18 and 45 years of age, non-smokers, not pregnant or breastfeeding, and free from acute and chronic diseases. Additional exclusion criteria included: no antibiotic use within the past four months; no disorders affecting the sense of smell, taste, or respiratory system; no multiple chemical sensitivity or zinc deficiency; no history of cranial trauma, concussion, or nasal polyps; and no history of chemo- or radiotherapy. Participants also had to be free from ongoing psychological treatment and demonstrate intact cognitive abilities, as indicated by a MoCA score of 26 or higher (M = 28.73, SD = 1.26, range = 26–30), show no clinically relevant depressive symptoms (BDI-II < 14; M = 3.17, SD = 3.53, range = 0–10), and achieve at least 11 points on the 16-item odor identification Sniffin’ Sticks test representing normal smell abilities (M = 13.07, SD = 1.60, range = 11–16). The study was approved by the Ethics Committee of the Medical Faculty of Friedrich-Alexander-Universität Erlangen-Nürnberg (ethics protocol 23-183-1-S). All participants provided written informed consent, and the study was conducted in accordance with the Declaration of Helsinki for medical research involving human subjects.

*Pilot: Experimental procedure*

Since the newly developed odor-color test is based on the color profile established for the MONEX-40^1^, a final set of 16 odors was selected from the original pool of 40 stimuli. To ensure methodological independence and to avoid potential legal and familiarity-related biases, the first 16 MONEX-40 odors—identical to those used in the patented 16-item Sniffin’ Sticks identification test—were excluded. In addition, only odors with a correct identification rate of at least 50% from the MONEX-40 color profile study were included in the new test. Odors associated with multivariate or ambiguous color associations (e.g., melon-bivariate: red/green) were also excluded to support a clear and interpretable odor-color mapping. Furthermore, odors with known allergenic potential (e.g., almond, peanut, coconut) were deliberately omitted to minimize health risks. To support the development of a mobile, user-friendly screening tool for future public use, we implemented a novel presentation format for the selected odors. The aim was to create a hygienic, intuitive, self-administered kit. Small brown glass jars (5 g; manufacturer: Benecreat, available via Amazon.de) were chosen as a recyclable and light-protective solution. Each jar was tightly sealed with a hygiene label and an additional airtight ring to ensure odor stability. To facilitate safe and stable integration of the odorants, we transitioned from the traditional liquid format (as used in MONEX-40 Sniffin’ Sticks) to a solid medium. The odorants were emulsified in Hard Fat W35 (Hard Fat/Adeps Solidus W35; manufacturer: IOI Oleo GmbH, Hamburg, Germany), chosen for its excellent emulsifying properties, solid consistency at room temperature, non-toxic profile, and minimal intrinsic odor. Each jar was filled with 1.5 ml of the respective stimulus, using a uniform tenfold reduction of the odors from the original MONEX-40 concentrations (Supplemental Table 1). The adjustment was made to standardize stimulus intensity across all odors, considering the new delivery format, which creates a lasting scent reservoir and positions the stimulus closer to the participant. An extensive pilot study was conducted to evaluate whether the novel odor presentation format using jars was comparable to the conventional Sniffin’ Sticks format. This step was essential, as the new odor-color test builds upon the color profile study^1^, which in turn was derived from the odor intensities of the Sniffin’ Sticks. For the pilot study, all individuals completed a set of questionnaires assessing exclusion criteria (see Section: Participants) and providing socio-demographic information. Given the known influence of depressive symptoms on olfactory performance^2^, participants also completed the Beck Depression Inventory-II (BDI-II). The BDI-II^3,4^ is a self-report questionnaire developed to measure the severity of depressive symptoms, as defined by the American Psychiatric Association’s Diagnostic and Statistical Manual of Mental Disorders, Fourth Edition (DSM-IV 1994). To minimize confounding variables, all participants were required to follow strict dietary and hygiene guidelines. These included abstaining from alcohol the evening before testing, and avoiding coffee, tobacco products, or medication for at least two hours prior to the session. On the day of testing, participants were instructed not to use any scented products such as perfumed creams, makeup, aftershave, deodorant, or perfume, to reduce external influences on olfactory perception. The testing procedure lasted approximately 60 minutes, with appointment slots offered throughout the day to counterbalance potential time-of-day effects and ensure an even distribution across participants. All sessions took place in a controlled sensory laboratory, under standardized lighting conditions with closed blinds. At the start of the session, participants completed the 16-item Sniffin’ Sticks identification test. Only those who scored at least 11 points—thus meeting the criteria for normosmia^5,6^—were allowed to proceed. These individuals then underwent cognitive screening using the standardized Montreal Cognitive Assessment (MoCA, version 2), a widely used tool for assessing various cognitive domains in neuropsychological research^7^. Participants scoring ≥ 26 points on the MoCA, indicating cognitive functioning within a healthy range, were admitted to the final pilot phase. During the pilot, each participant was presented with both stimulus formats: the traditional Sniffin’ Sticks (applied to paper, as in the MONEX-40 color-profile study) and the newly developed odor jars. The presentation order of both the odor types (Sniffin’ Stick versus jar) and the individual odor IDs was fully randomized. Each odor was held approximately one cm below the nose by the experimenter. Participants rated all odors on three dimensions—intensity, pleasantness, and familiarity—using a simplified visual analog scale (VAS) ranging from 0 to 10 (intensity (0 = no odor, 10 = very intense), pleasantness (0 = very unpleasant, 10 = very pleasant), familiarity (0 = not familiar, 10 = very familiar). Responses were entered directly by the participants using a 10th generation Apple iPad (2022) with full display brightness and no screen filters. A 30-second interval was maintained between each stimulus to minimize olfactory adaptation or cross-sample effects. Participants received a compensation of €10 for their participation, which lasted approximately 60 minutes.

**Supplemental Table 1. Composition of odor stimuli in the preliminary study and the final test.** The table lists the 16 final odors along with their respective concentrations. In the color-profile study, Sniffin’ Sticks were used with either propylene glycol (PG) or diethyl phthalate (DEP) as the carrier medium. In contrast, the stimuli in the newly developed odor-color test were uniformly reduced in concentration by a factor of ten, emulsified in hard fat, and filled into small jars. Odor ID No. 17 was used exclusively in the pilot study as a blank control.

*Statistical analysis*

For the analysis of the pilot study, mean values and standard errors of the mean (SEM) for intensity, familiarity, and pleasantness were descriptively compared between the Sniffin’ Sticks and the new jar-based odor presentations. Visualizations were created using Biorender.com.

*Results*

The results clearly show that each individual odor was perceived as at least equally intense as in the preliminary study using Sniffin’ Sticks (Supplemental Table 2). This was a key objective of the new presentation format, as the congruent and incongruent colors used in the newly developed odor-color test are based on the color profile and intensity ratings established in the MONEX-40 study. In addition, ratings of familiarity and pleasantness were collected and found to be comparable across presentation formats, although these measures were not the primary focus of the evaluation (Supplemental Figure 1).

**Supplemental Table 2. Descriptive analysis of the pilot study: comparison of odor presentation formats.** Descriptive statistics for intensity (I), familiarity (F), and pleasantness (P) across all odors (1–16) and the blank odor condition (17), separately reported for each presentation format: Jar (“J”) and Sniffin’ Stick (“S”)


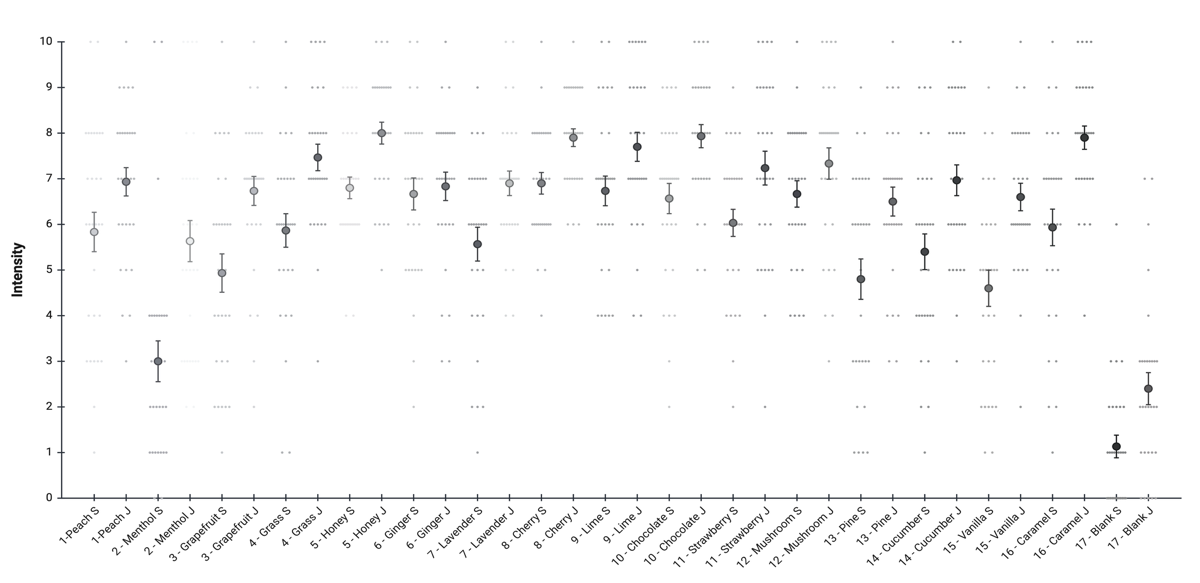

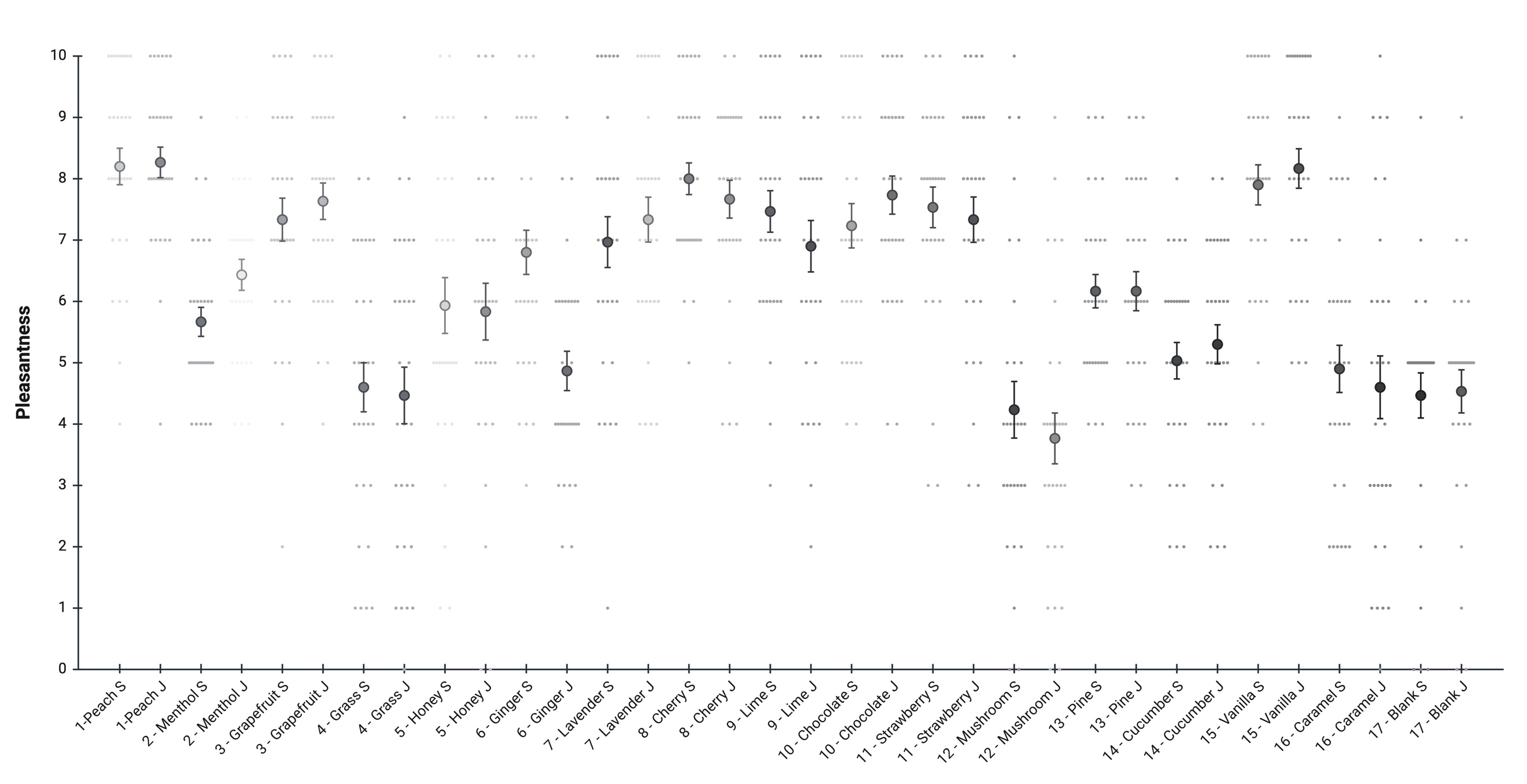

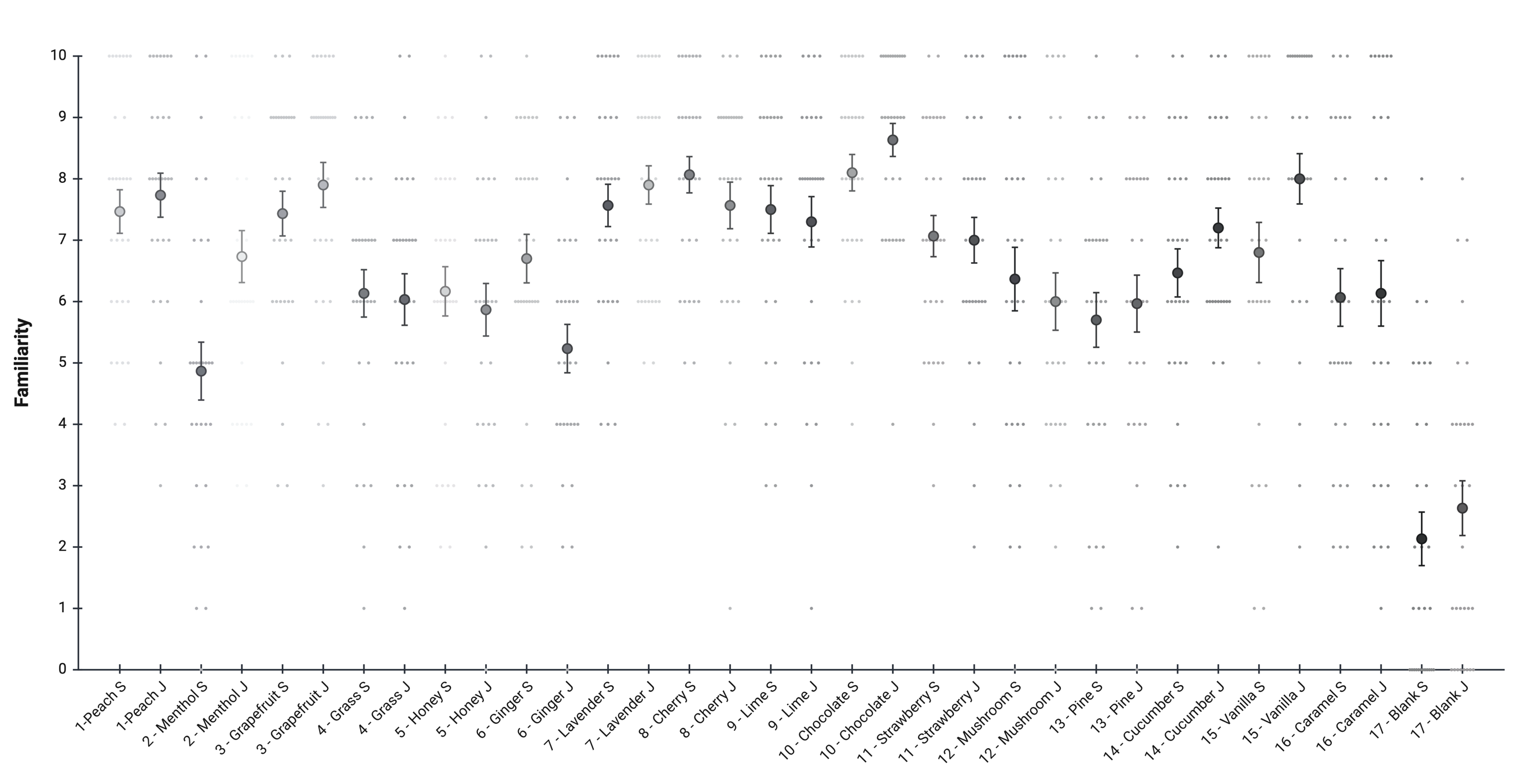


**Supplemental Figure 1. Pilot odor ratings by presentation format.** Mean (circle) ratings for perceived intensity, familiarity, and pleasantness across odors, with standard error of the mean (SEM) shown as error bars. Crucially, all odors presented in jars (J) were perceived as at least equally intense as their Sniffin’ Stick (S) counterparts, supporting their use in the main experiment. Familiarity and pleasantness ratings were comparable across formats, indicating consistent recognition and affective evaluation regardless of delivery method.

**Supplemental Material 2: Detailed demographic profile**


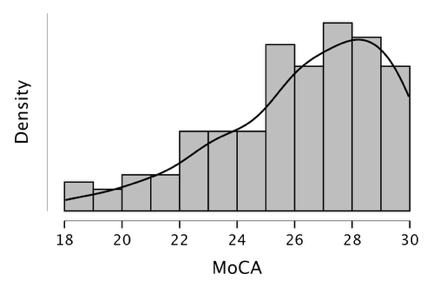

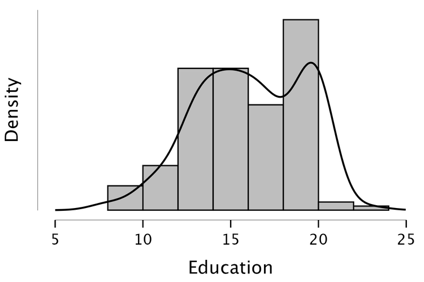

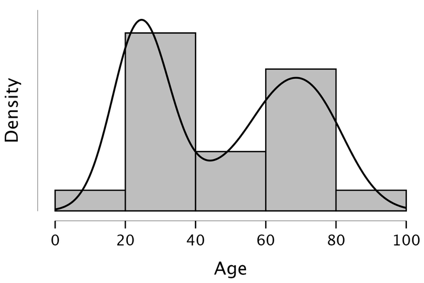


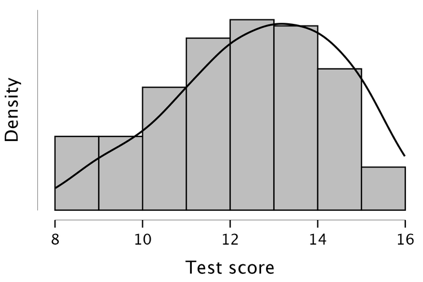

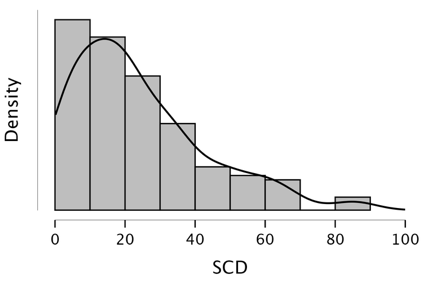

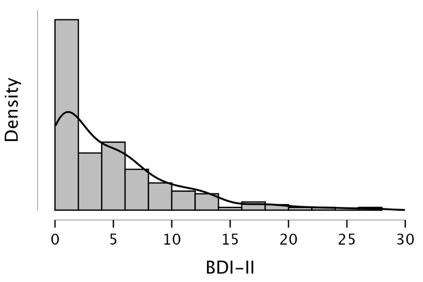


**Supplemental Figure 2. Distribution plots.** Distribution plots of the variables Age, Education, MoCA, BDI-II, SCD and Test score.

**Supplemental Material 3: Detailed experimental procedures**

*Color vision screening protocol*

Color vision was screened using a digital version of the Kuchenbecker-Broschmann color charts^8^, displayed on a standardized tablet. Participants viewed the charts from approximately 70 cm at a fixed angle and were asked to identify one to three embedded numbers or letters within 15 seconds (Supplemental Figure 3). The following charts were used: Chart 6R and Chart 25 (for red-green deficiency), and Chart 49 (for blue-yellow deficiency). Participants had to correctly identify all three to proceed with the study. Participants wearing visual aids with optical filters (e.g., blue-light filters) were excluded unless they could perform the test with non-filtered glasses or without any aid. Red-green deficiencies were screened carefully, given their prevalence (~8% of men and ~0.4% of women in Europe^9^), whereas blue-yellow deficiencies, being extremely rare, were included primarily for completeness^10^.


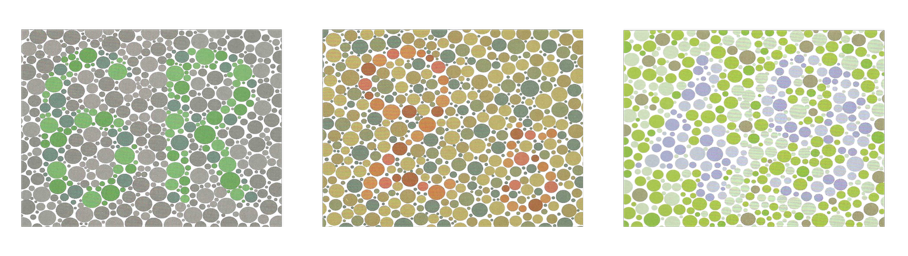


**Supplemental Figure 3. Color vision screening tool.** From left to right: "6R" and "25" (for testing red-green deficiency), "49" (for testing blue-yellow deficiency). Based on the color charts of Kuchenbecker & Broschmann^8^.

*Test platform and device specifications*

The odor-color test was delivered via a web-based application hosted on PythonAnywhere.com. All participants used identical devices: Lenovo Tab M10 FHD Plus, 2nd Generation (model TB-X606F), running Android 10. Display settings were standardized across all sessions: maximum screen brightness, font size, and display zoom were enabled, and all visual filters (e.g., blue-light filters, night mode) were disabled to ensure consistent color presentation. The test application ran in full-screen mode to minimize distractions and visual inconsistencies.

*Translation procedure for the SCD inventory*

The Subjective Cognitive Decline (SCD) questionnaire (McCusker version^11^) was translated into German following standard back-translation methodology. Initially, two native German speakers independently translated the original English version. These translations were reviewed and harmonized into a unified draft. A native English speaker then back-translated the German version into English. The back-translated version was compared to the original, and discrepancies were discussed and resolved in consultation with a bilingual researcher to ensure semantic and conceptual equivalence.

**Supplemental Material 4: Control analyses ≥ 60 years**

**Supplemental Table 4. Statistical comparisons of test scores between congruent and incongruent conditions in participants aged 60 and above.** The first table presents statistical comparisons of the variables age, education, MoCA, and test score between the congruent and incongruent versions within the group of interest (participants aged 60+). Tests for normality (Shapiro–Wilk) and equality of variances (Levene’s test) were conducted to determine appropriate test selection. The lower table summarizes binary group comparisons between the two test versions in this age group with respect to test setting (lab room versus real-life environment), sex, smoking status, and categorical groupings of self-reported diseases and medication profiles (based on sociodemographic responses). Depending on expected cell sizes, either Chi-square or Fisher’s exact test was applied. Two optional correction methods for multiple comparisons (Bonferroni and FDR) were included. Since no significant differences were found in age, education, MoCA, test setting, sex, smoking, or medical/medication profiles, the significant difference in test score is likely attributable to differences in cross-modal processing of color congruency.


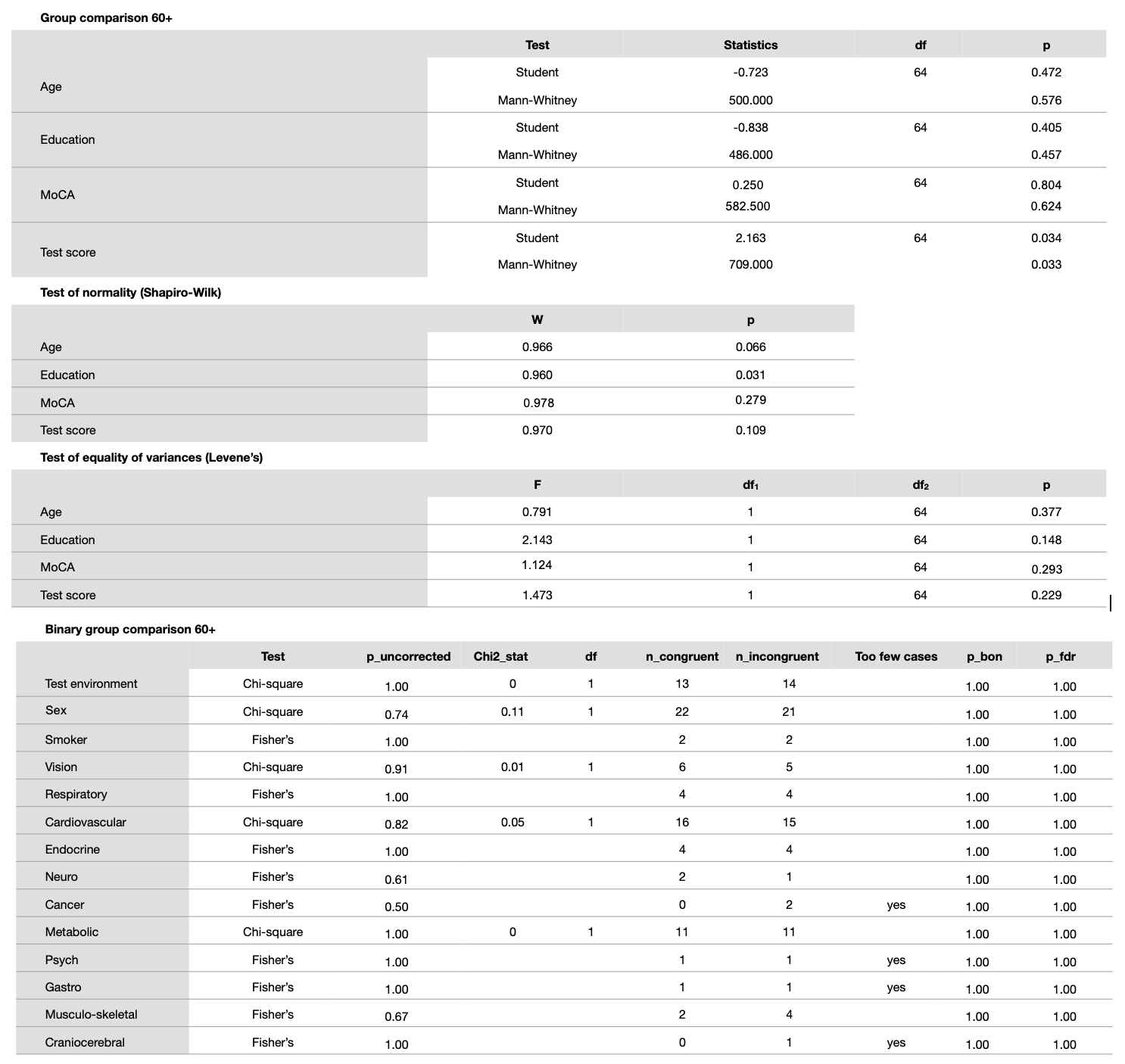


**Supplemental Material 5: Supplementary plots and regression analyses**

**Predictive regression results**

Test score vs. Age Congruent: β = -3.49, 95% CI [-5.81, -1.17], p = 0.004, R² = 0.10; Incongruent: β = -6.60, 95% CI [-8.40, -4.82], p < 0.001, R² = 0.30

Test score vs. MoCA Congruent: β = 0.60, 95% CI [0.22, 0.97], p = 0.002, R² = 0.15; Incongruent: β = 0.76, 95% CI [0.49, 1.03], p < 0.001, R² = 0.26

Mean Intensity vs. Age Congruent: β = 0.18, 95% CI [-4.50, 4.84], p = 0.94, R² = 0.00062; Incongruent: β = -5.12, 95% CI [-10.08, -0.16], p = 0.04, R² = 0.06

Mean Intensity vs. MoCA Congruent: β = -0.25, 95% CI [-0.95, 0.45], p = 0.48, R² = 0.006; Incongruent: β = 0.23, 95% CI [-0.37, 0.83], p = 0.44, R² = 0.007

Mean Pleasantness vs. Age Congruent: β = 3.46, 95% CI [-1.16, 8.07], p = 0.14, R² = 0.03; Incongruent: β = -3.54, 95% CI [-8.73, 1.64], p = 0.12, R² = 0.03

Mean Pleasantness vs. MoCA Congruent: β = -0.34, 95% CI [-0.98, 0.30], p = 0.28, R² = 0.01; Incongruent: β = 0.28, 95% CI [-0.34, 0.90], p = 0.37, R² = 0.01

Mean Familiarity vs. Age Congruent: β = -19.45, 95% CI [-50.96, 12.06], p = 0.22, R² = 0.02; Incongruent: β = -53.34, 95% CI [-77.05, -29.63], p < 0.001, R² = 0.14

Mean Familiarity vs. MoCA Congruent: β = 1.54, 95% CI [-4.38, 7.48], p = 0.61, R² = 0.007; Incongruent: β = 3.21, 95% CI [-0.77, 7.21], p = 0.11, R² = 0.03

Test score vs. SCD: Congruent: β = -2.25, 95% CI [-4.45, -0.05], p = 0.045, R² = 0.05; Incongruent: β = -2.27, 95% CI [-4.36, -0.17], p = 0.03, R² = 0.05

Age vs. MoCA: Congruent: β = -0.08, 95% CI [–0.10, –0.05], p < 0.001, R² = 0.33; Incongruent: β = -0.07, 95% CI [–0.09, –0.05], p < 0.001, R² = 0.34

Test score vs. BDI-II: Congruent: β = -0.12, 95% CI [-0.67, 0.43], p = 0.67, R² = 0.002; Incongruent: β = 0.16, 95% CI -0.50, 0.83], p = 0.63, R² = 0.003

Mean Intensity vs. Mean Pleasantness: Congruent: β = 0.42, 95% CI [0.21, 0.64], p < 0.001, R² = 0.16; Incongruent: β = 0.40, 95% CI [0.20, 0.60], p < 0.001, R² = 0.17


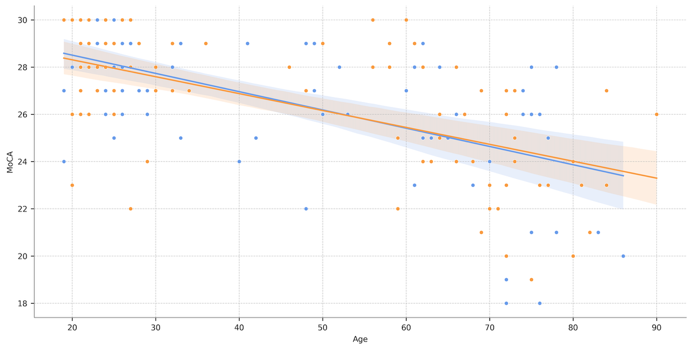
**
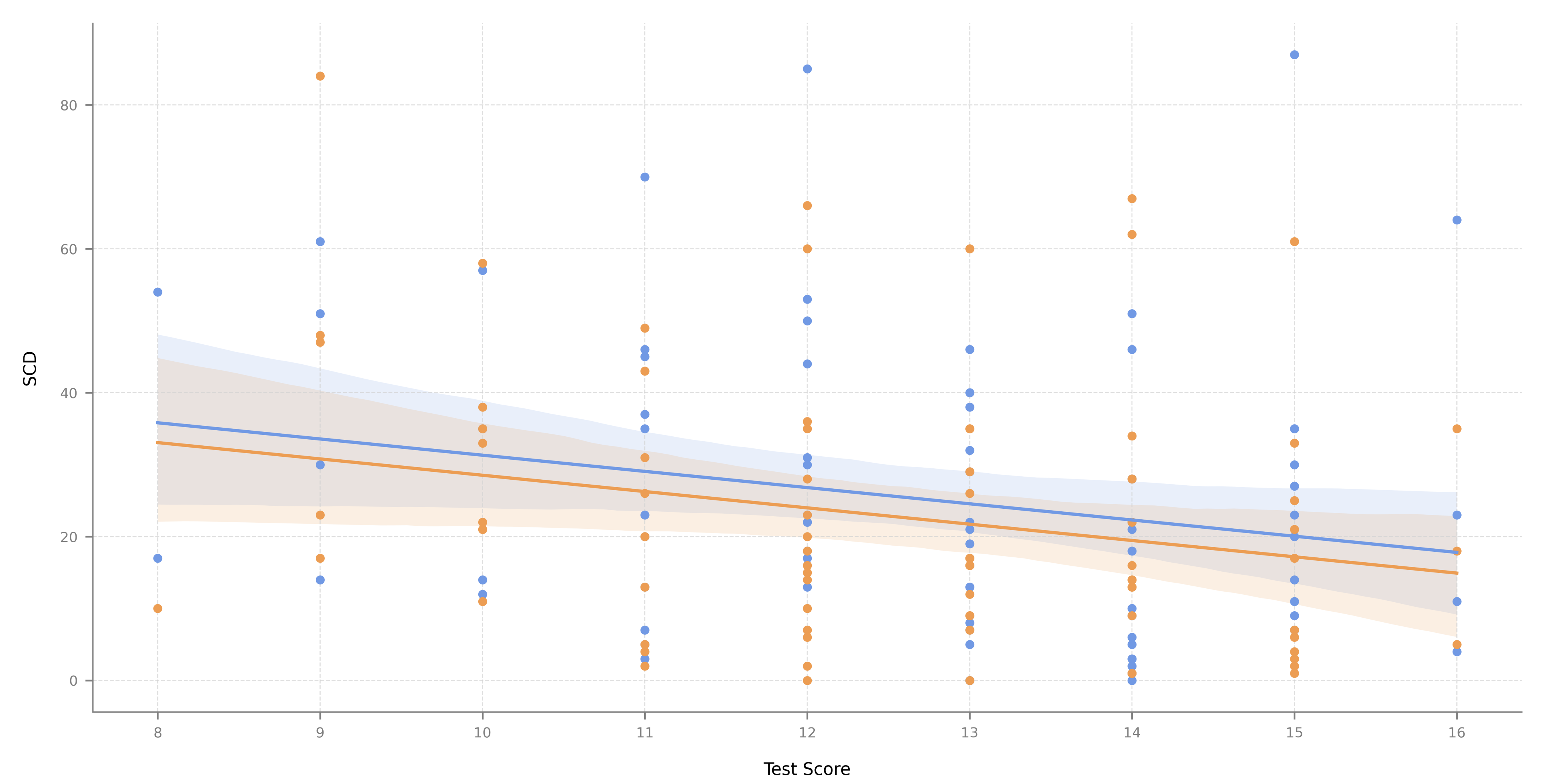
**

Congruent rho = -0.56, p = < 0.001

Incongruent: rho = -0.57, p = < 0.001

z = 0.10, p = 0.92

Congruent: rho = -0.24, p = 0.03

Incongruent: rho = -0.24, p = 0.03

z = 0.02, p = 0.98

MoCA

SCD

Age

Test score

***

***

*

*

**
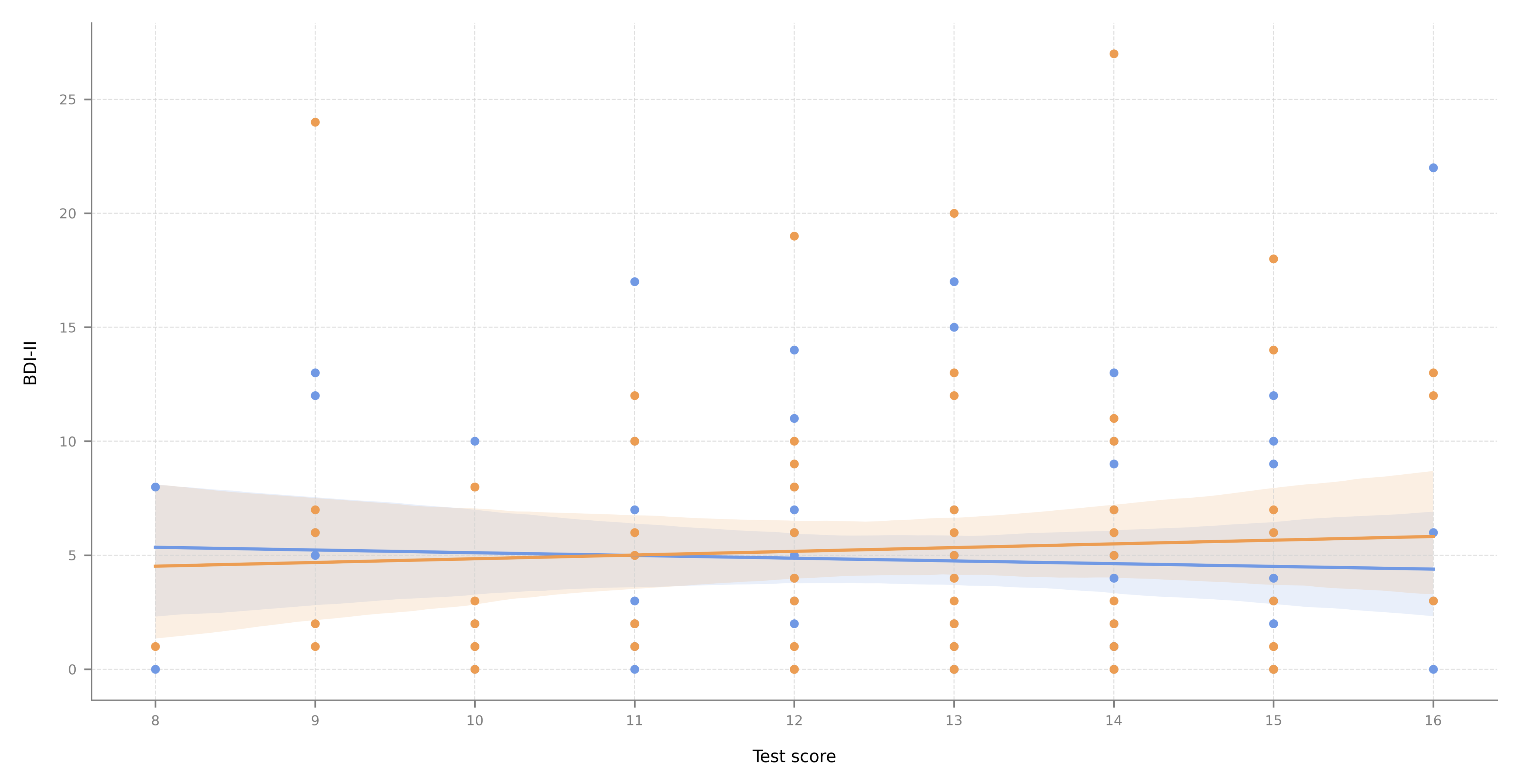
**
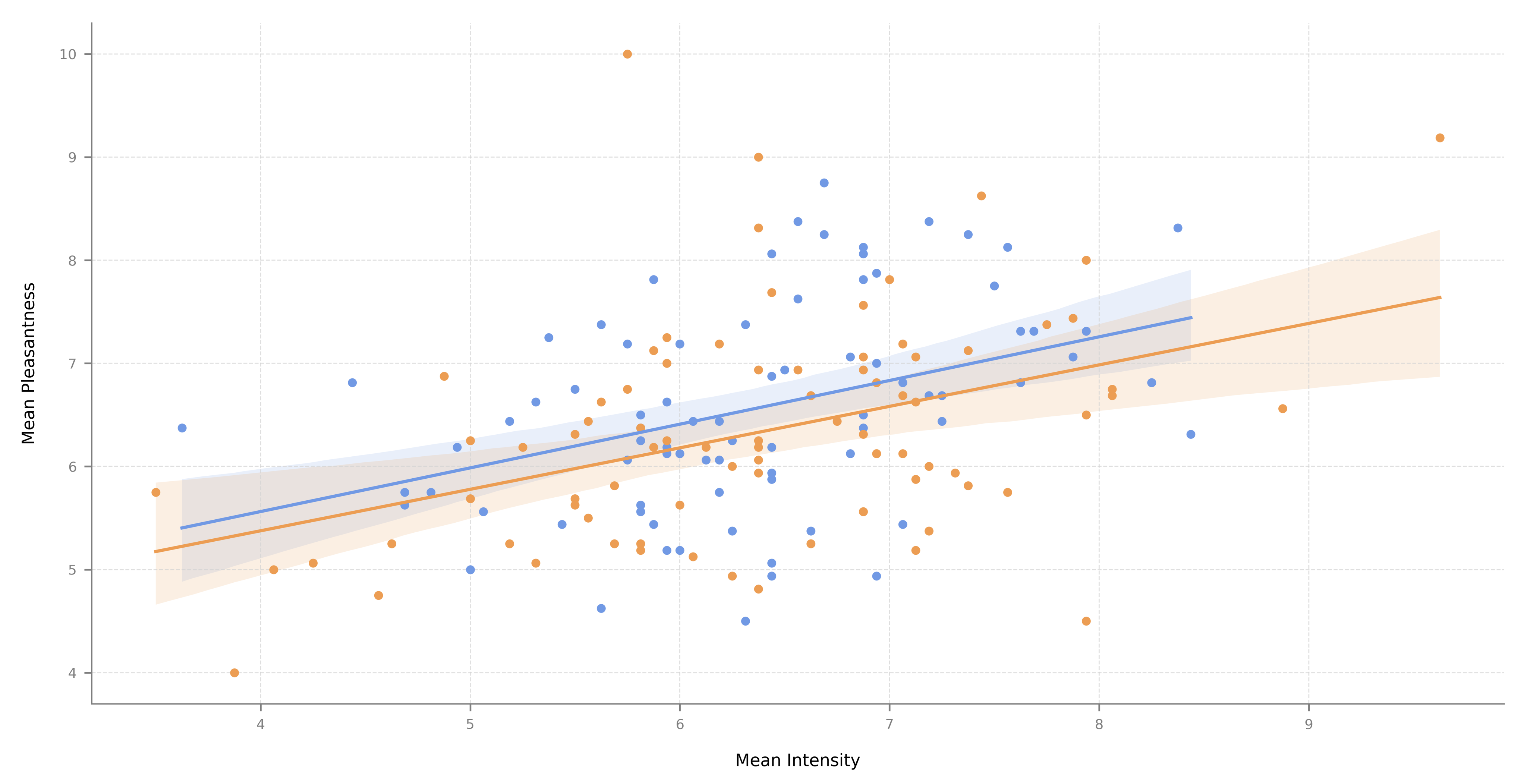


Congruent: r = 0.40, p = < 0.001

Incongruent: rho = 0.38, p = < 0.001

z = 0.16, p = 0.87

Congruent: rho = -0.08, p = 0.48

Incongruent: rho = 0.01, p = 0.91

z = -0.58, p = 0.56

Mean Intensity

Mean Pleasantness

BDI-II

Test score

***

***

**Supplemental Figure 4. Correlation and regression analyses across key variables.** Scatterplots depict relationships between test score and subjective cognitive decline (SCD), age and MoCA, test score and BDI-II, and mean intensity and mean pleasantness ratings. Trend lines are shown separately for the congruent (blue) and incongruent (orange) versions, each surrounded by shaded 95% confidence intervals. Asterisks indicate the significance of correlation coefficients (p-values).

**Supplemental Material 6: Generalized Linear Mixed Model (GLMM)**


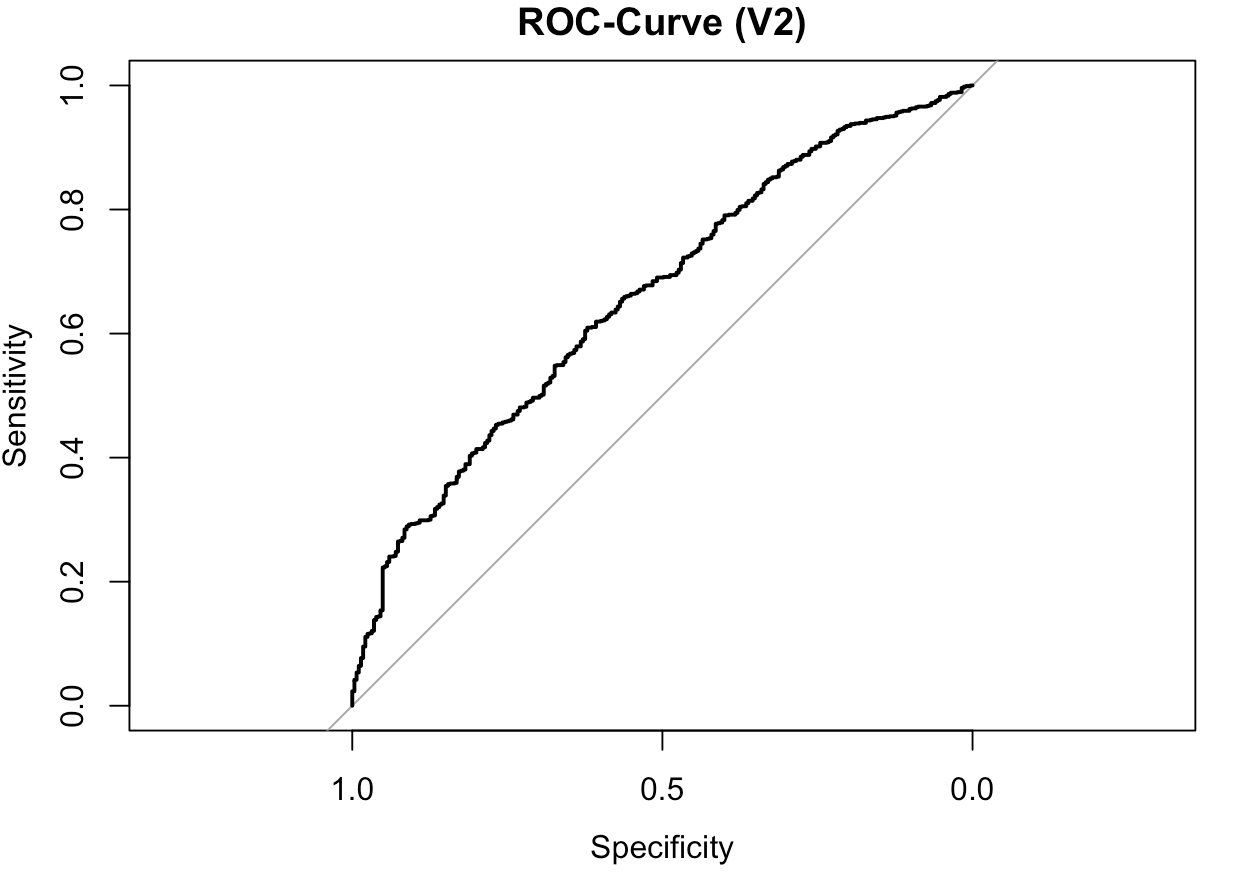


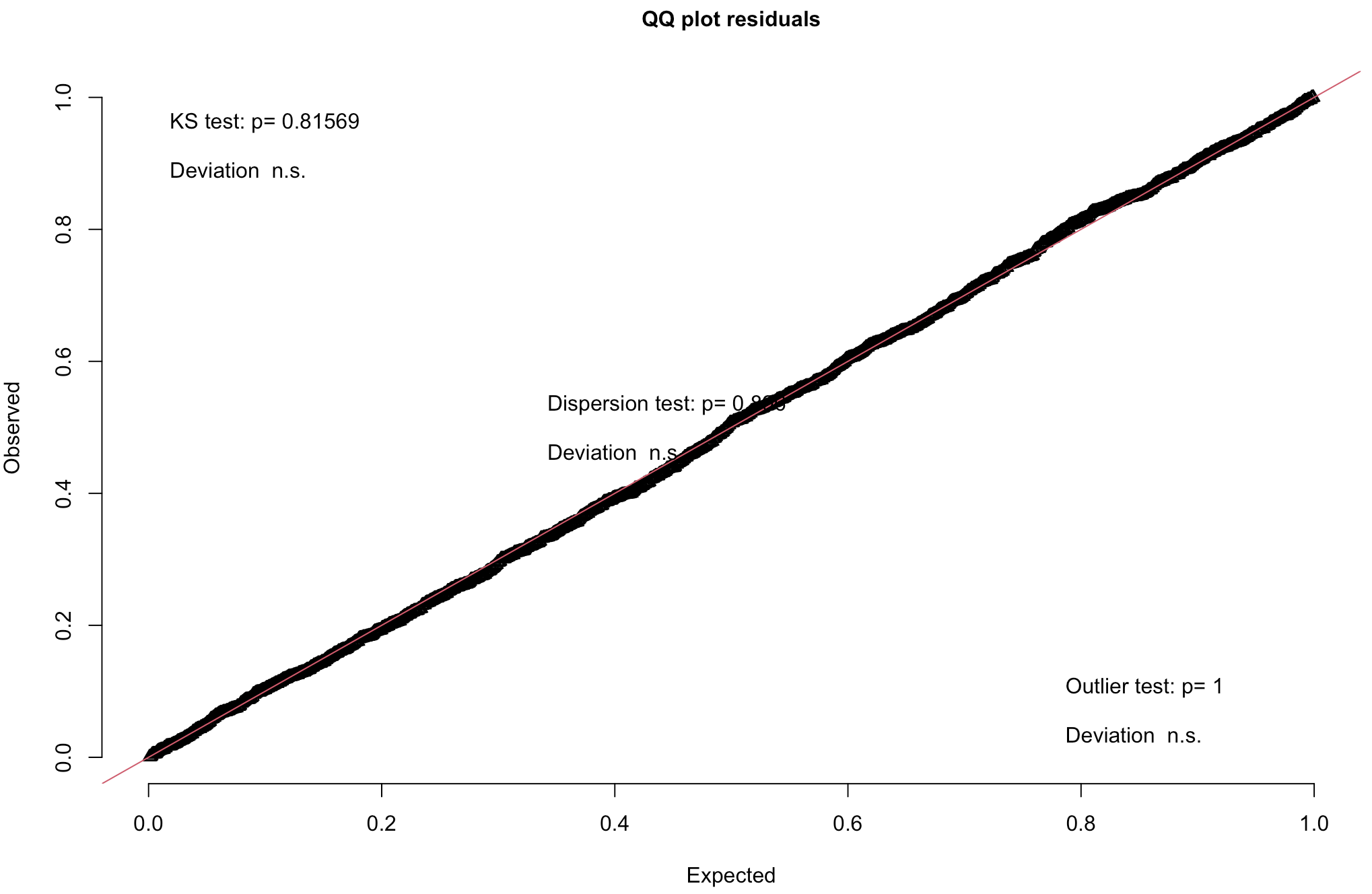


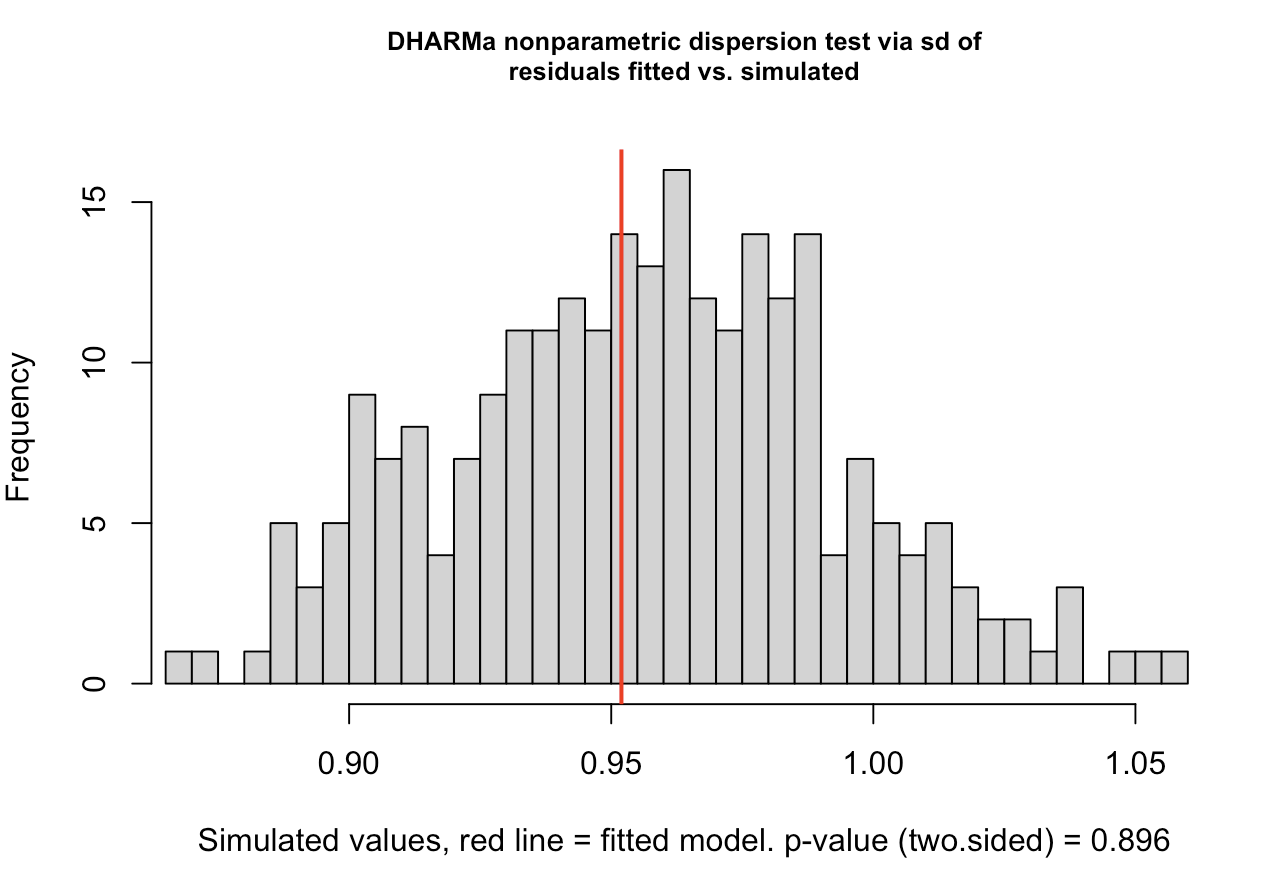


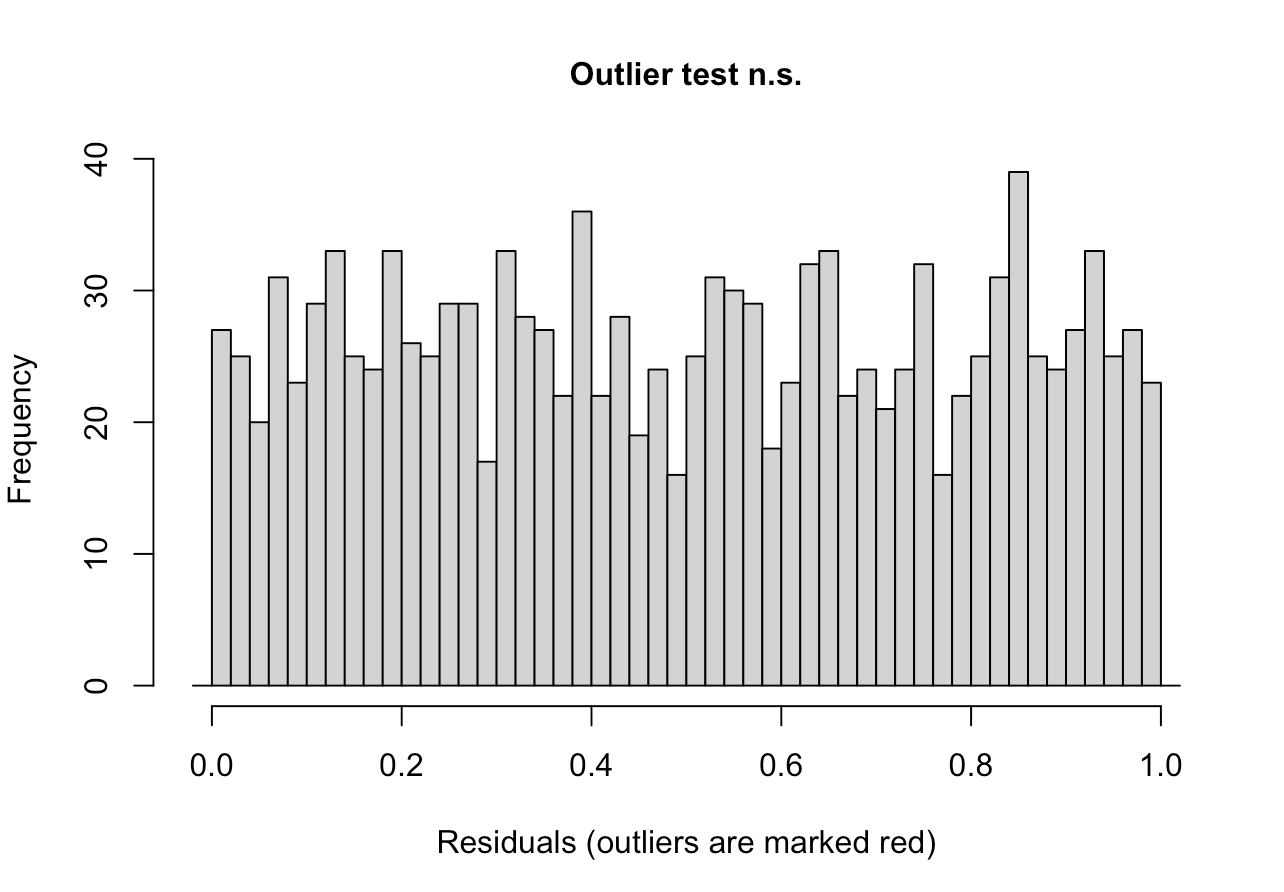


**Supplemental Figure 5. Model evaluation including ROC curve and DHARMs diagnostics.** From top to bottom: ROC curve illustrating the model’s ability to discriminate between correct and incorrect responses, with an area under the curve (AUC) of 0.66 indicating moderate predictive performance; QQ plot assessing the distribution of simulated residuals to check for deviations from uniformity; dispersion test evaluating whether the model shows signs of over- or under-dispersion; and outlier test detecting influential data points that could affect model stability. Together, these diagnostics support the model’s adequacy and validity.

*Generalized linear mixed model: age-stratified analyses*

To assess potential age-related differences, the model was additionally estimated separately for younger (<60 years) and older participants (≥60 years) participants. Both age-specific models revealed a non-linear association between color difference (∆ECIE2000) and response accuracy, using cubic splines. In the younger group (<60), all three spline components were statistically significant: first (p=0.024), second (p=0.008), third (p=0.003), indicating a non-linear relationship. In the older participants (≥60), a similar but slightly attenuated pattern emerged: the second (p<0.001) and third (p=0.01) spline terms were significant, while the first spline term showed only marginal significance (p=0.09). The effect of MoCA was positive in both models but did not reach statistical significance. However, both stratified models exhibited singular fits with zero random effect variance, indicating limited between-subject variability and reduced model stability. Furthermore, predictive performance was lower in the stratified models (AUC=0.61 for <60; AUC=0.63 for ≥60) compared to the full model. Due to these stability concerns and the superior overall model fit in the full sample (AUC=0.66; non-singular), all reported results in the main manuscript refer to the model based on the complete (all ages) incongruent dataset.

**Supplemental Table 5. Results of model including bs(ECIE2000_mod) and MoCA as predictors.** Displayed are the regression coefficients (Estimate), standard errors (Std. Error), z-values, and associated p-values.


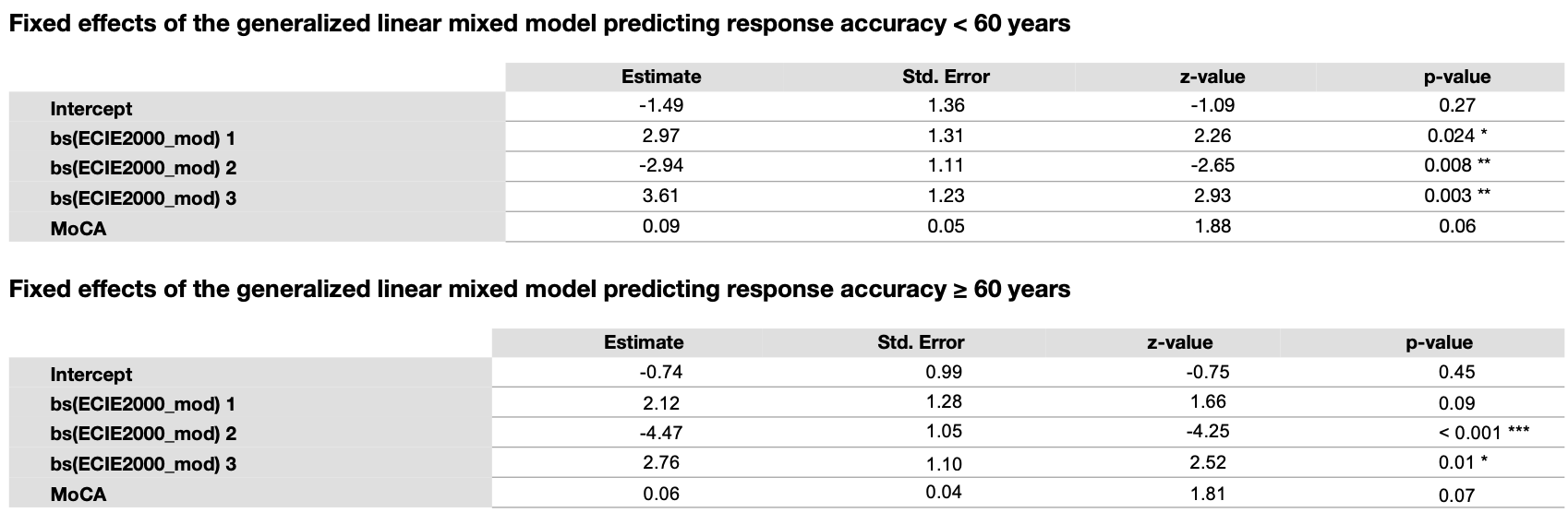


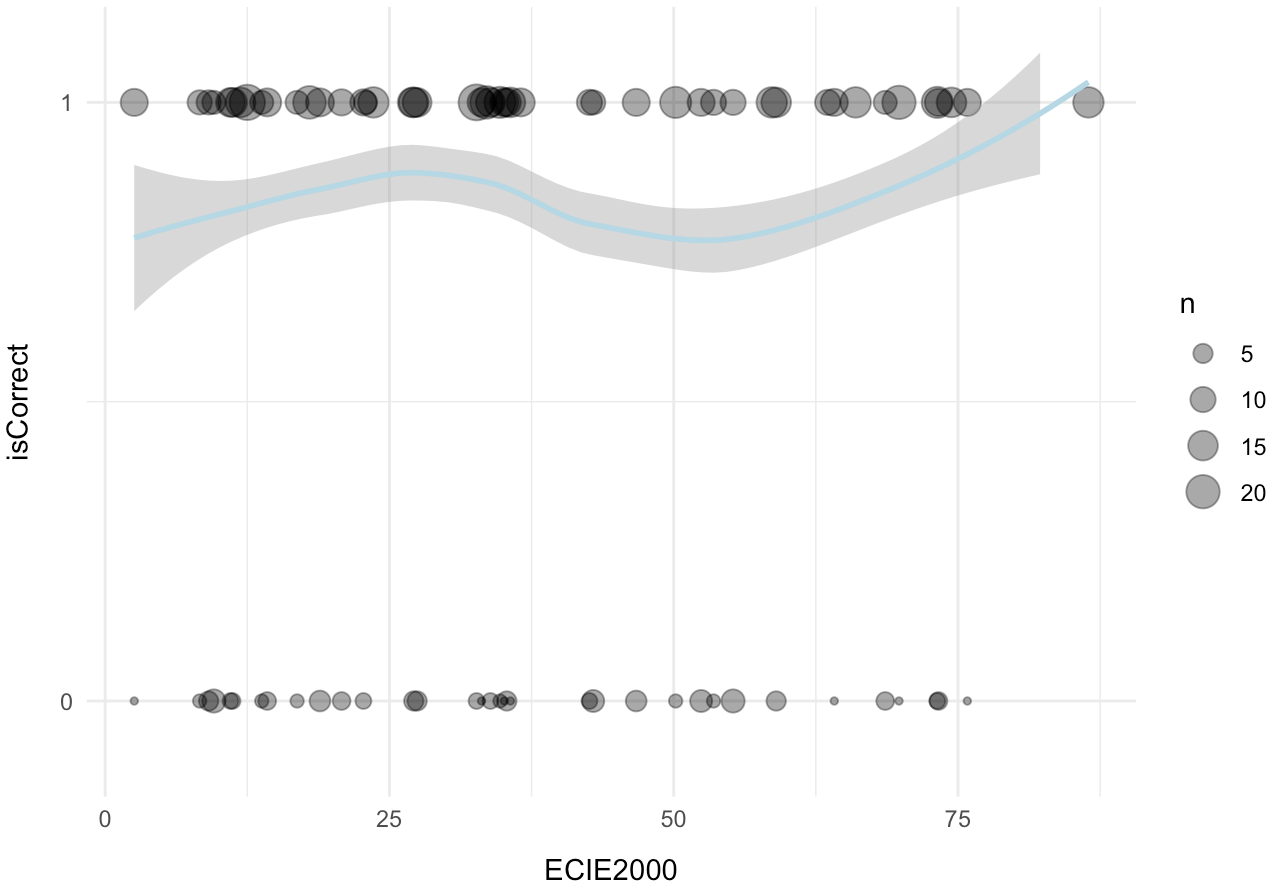

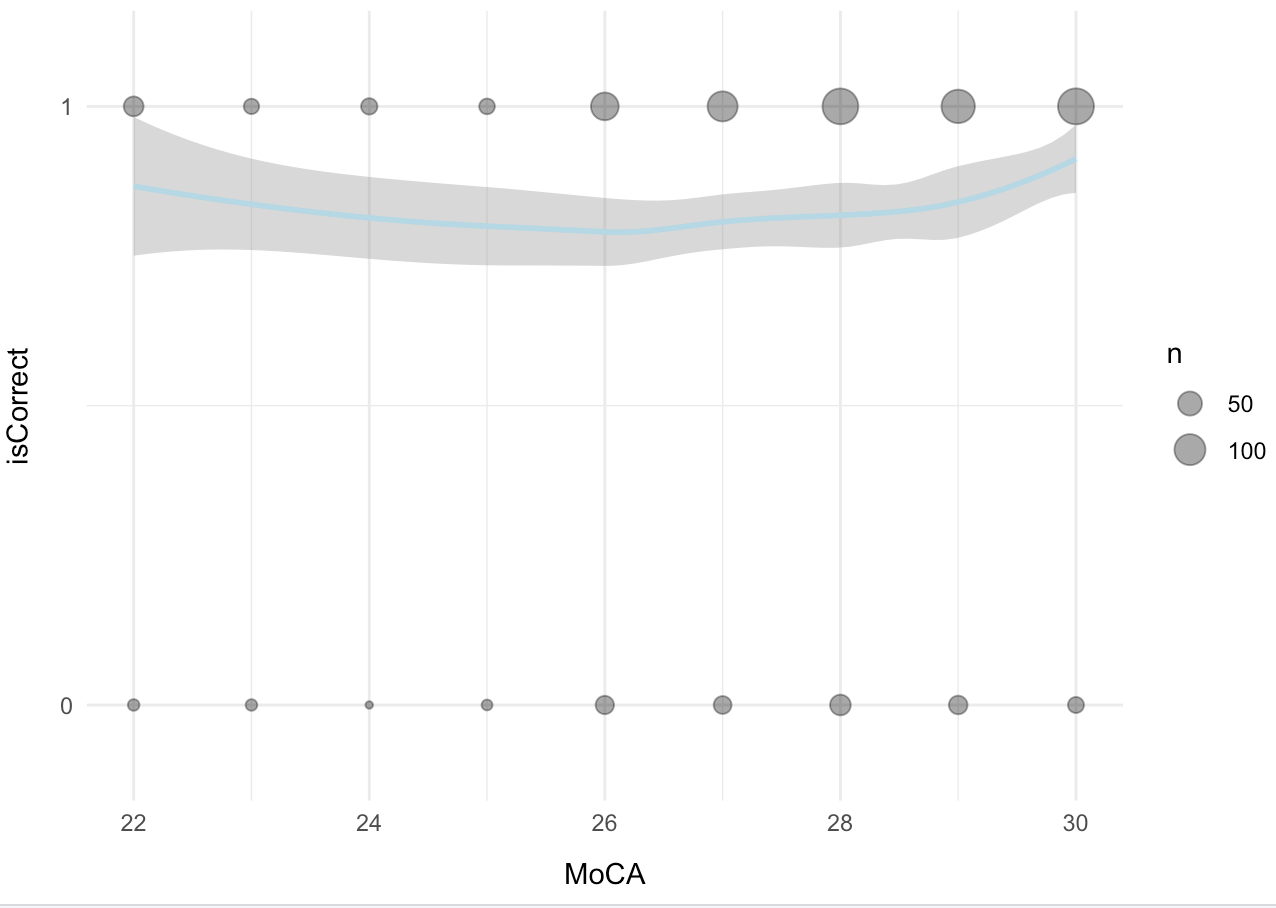


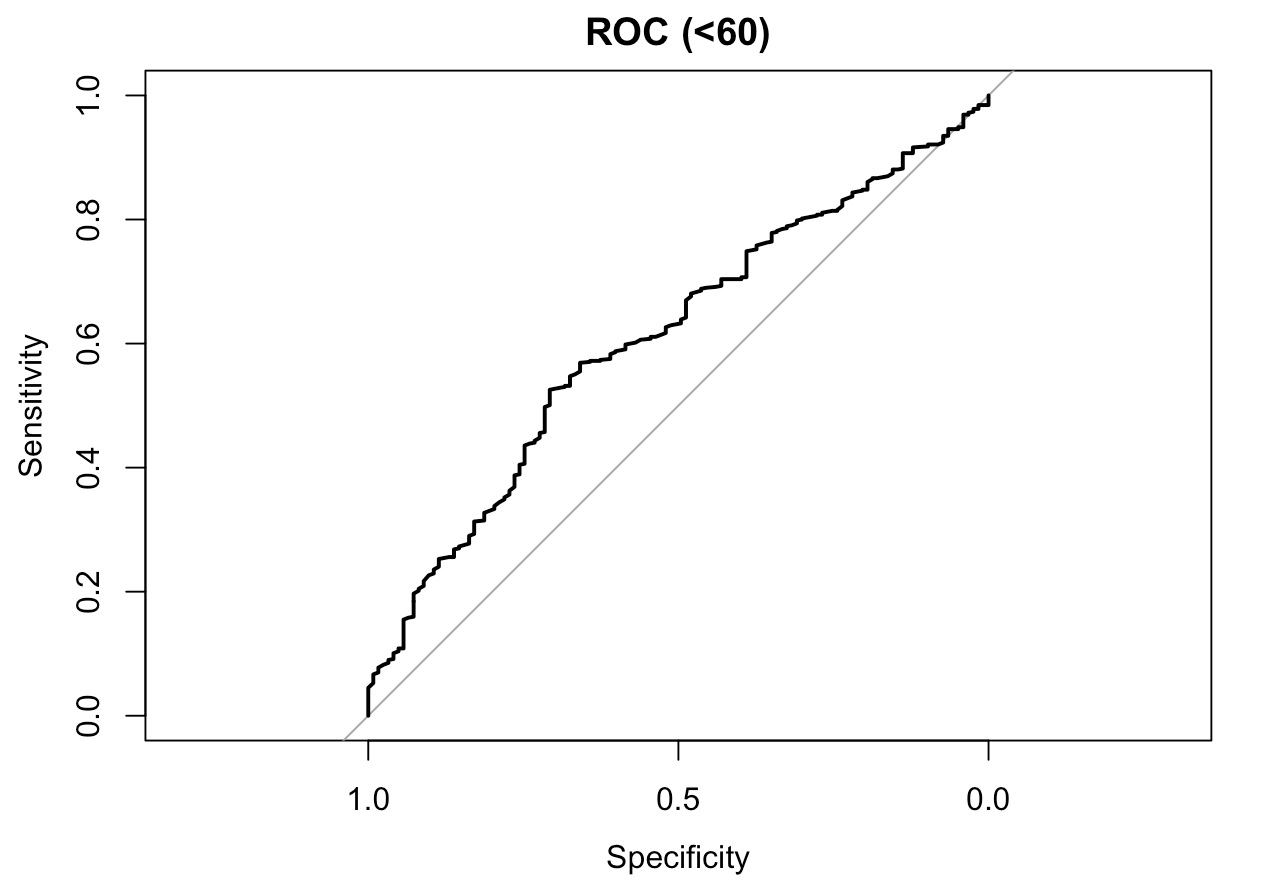

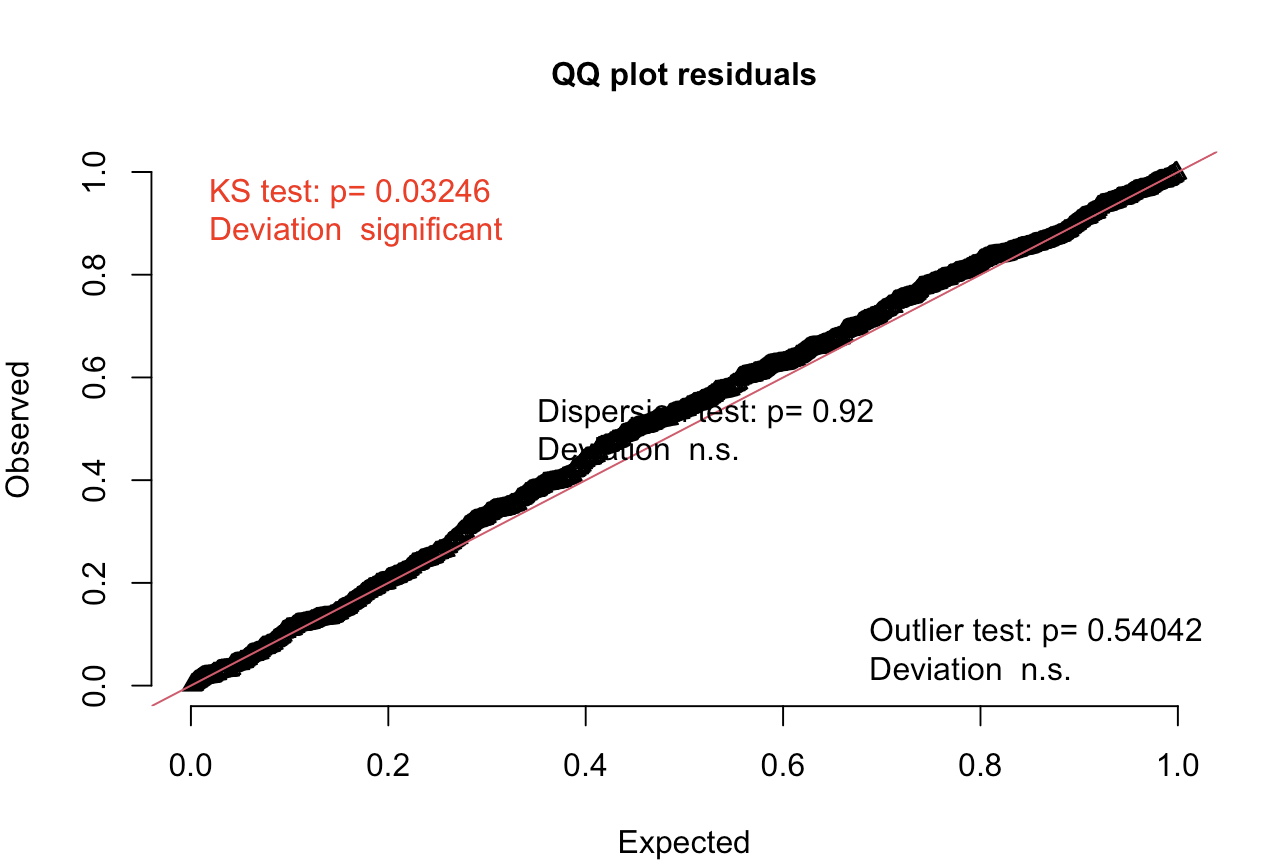


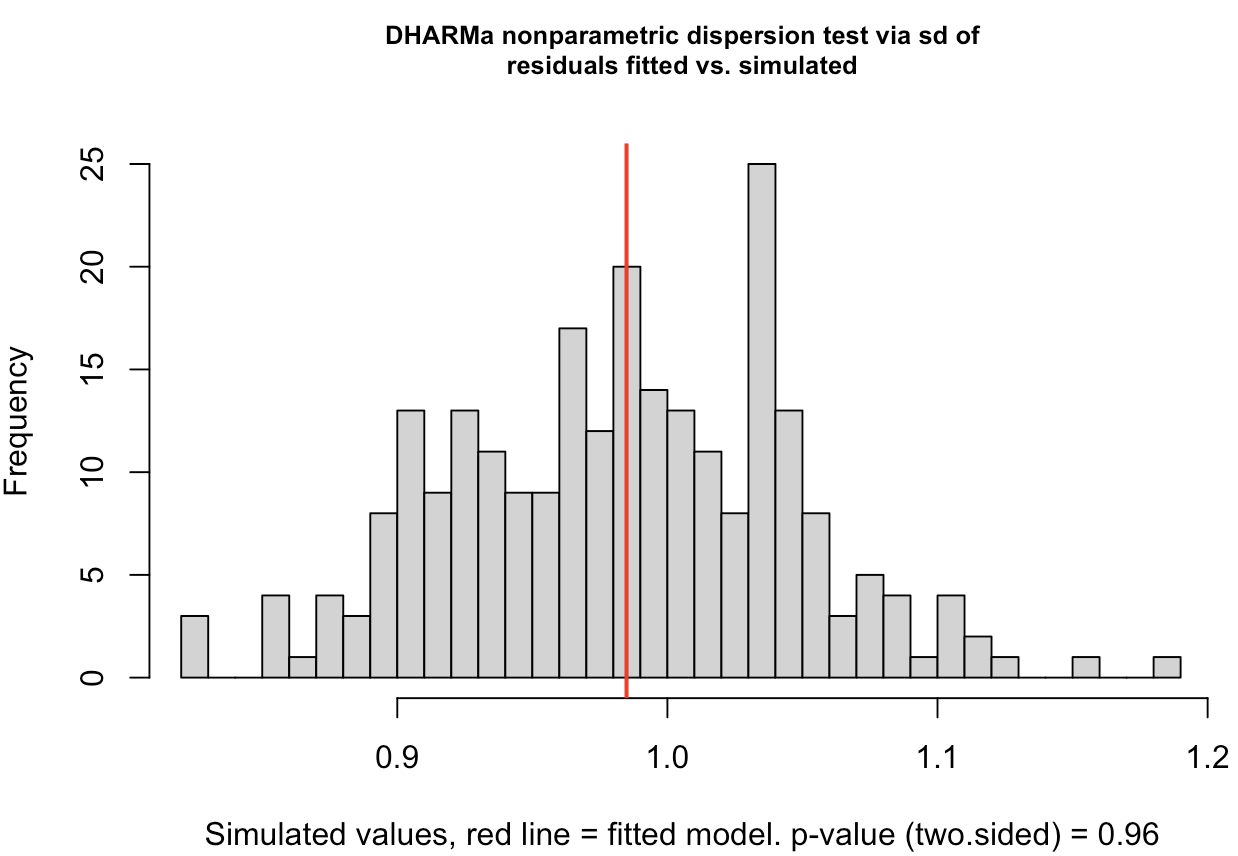

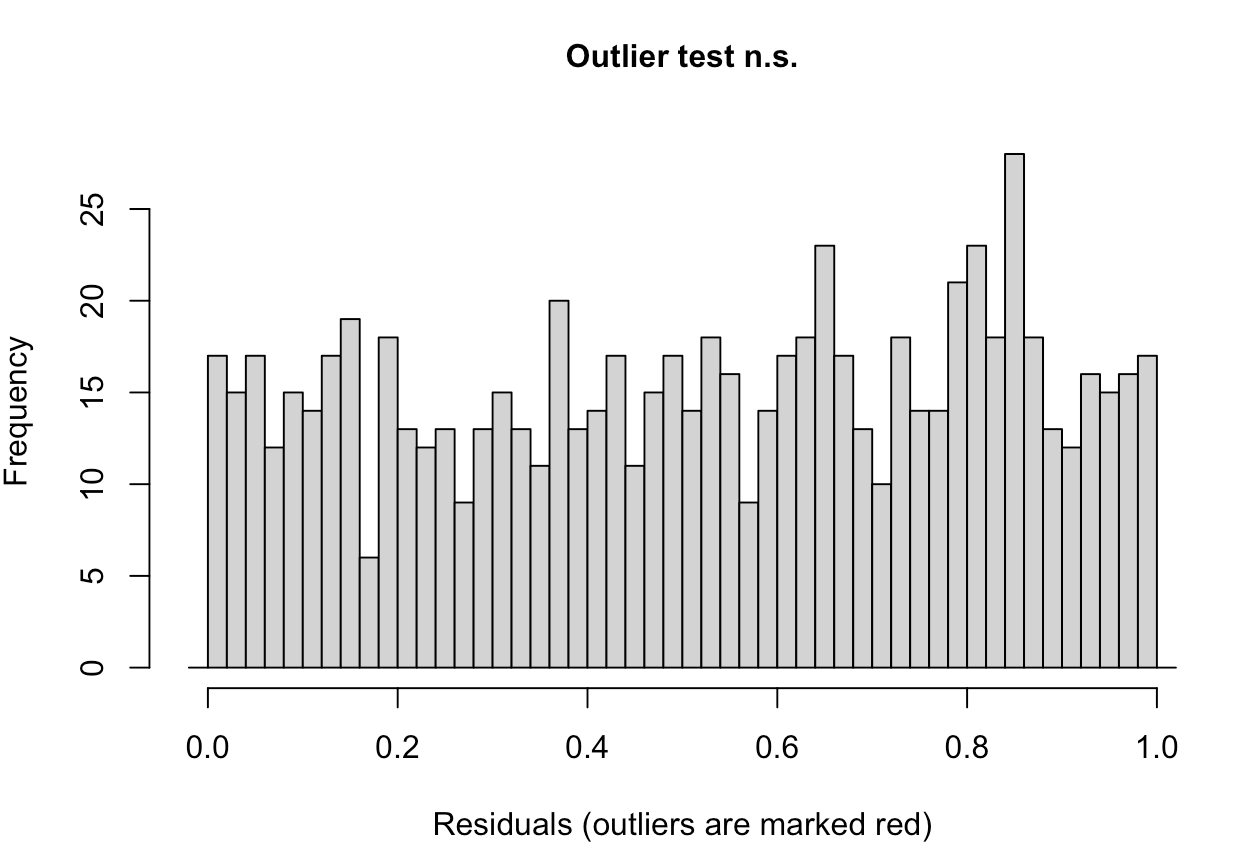


**Supplemental Figure 6. Overview of model predictions and diagnostics for participants < 60 years.** Top left: relationship between correct responses (isCorrect) and ΔECIE2000. Top right: relationship between correct responses and MoCA scores. Middle left: ROC curve assessing model discrimination. Middle right: DHARMa residual diagnostics checking for deviations from uniformity. Bottom left: DHARMa dispersion test assessing over- or under-dispersion. Bottom right: DHARMa outlier test identifying influential observations.


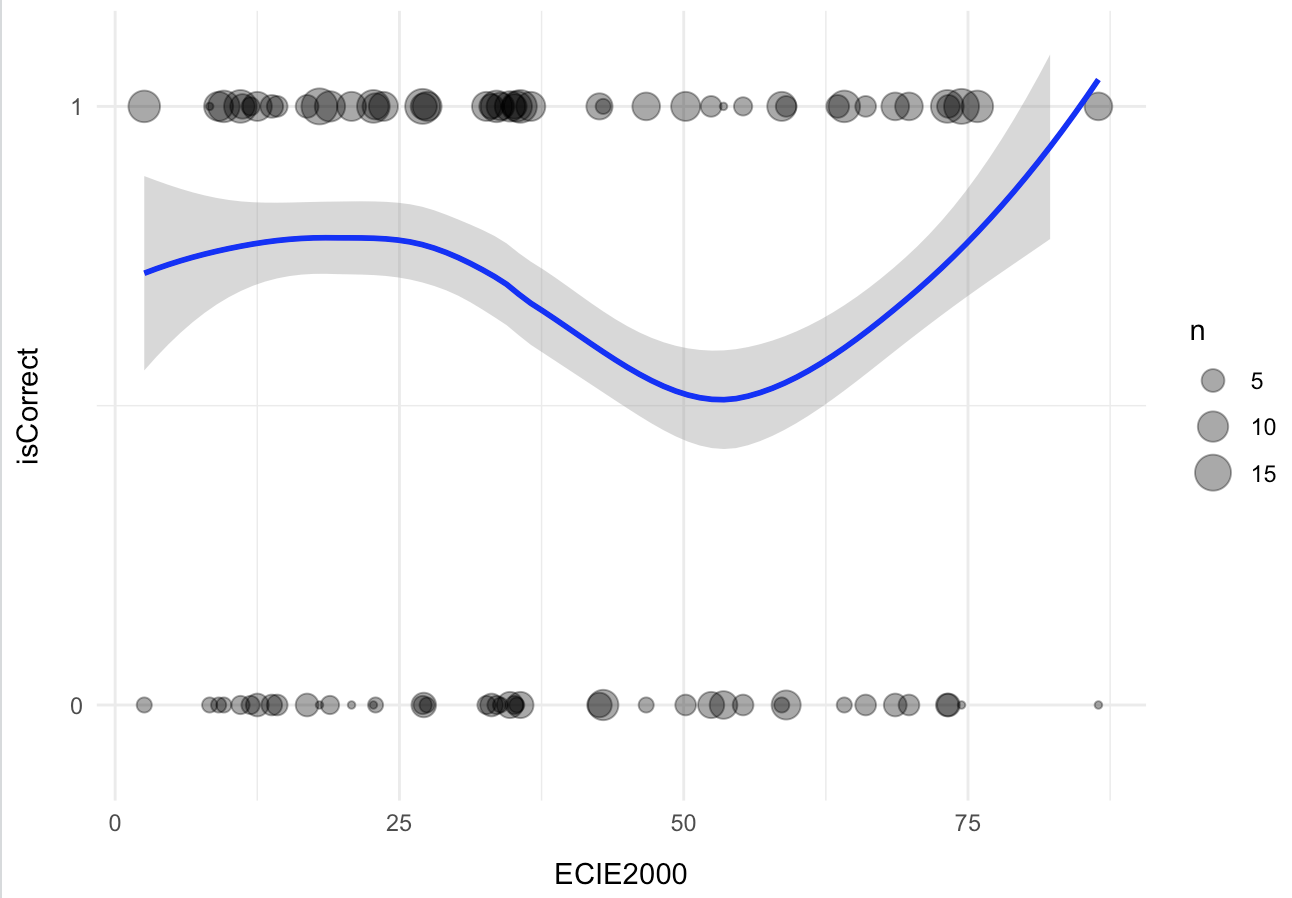

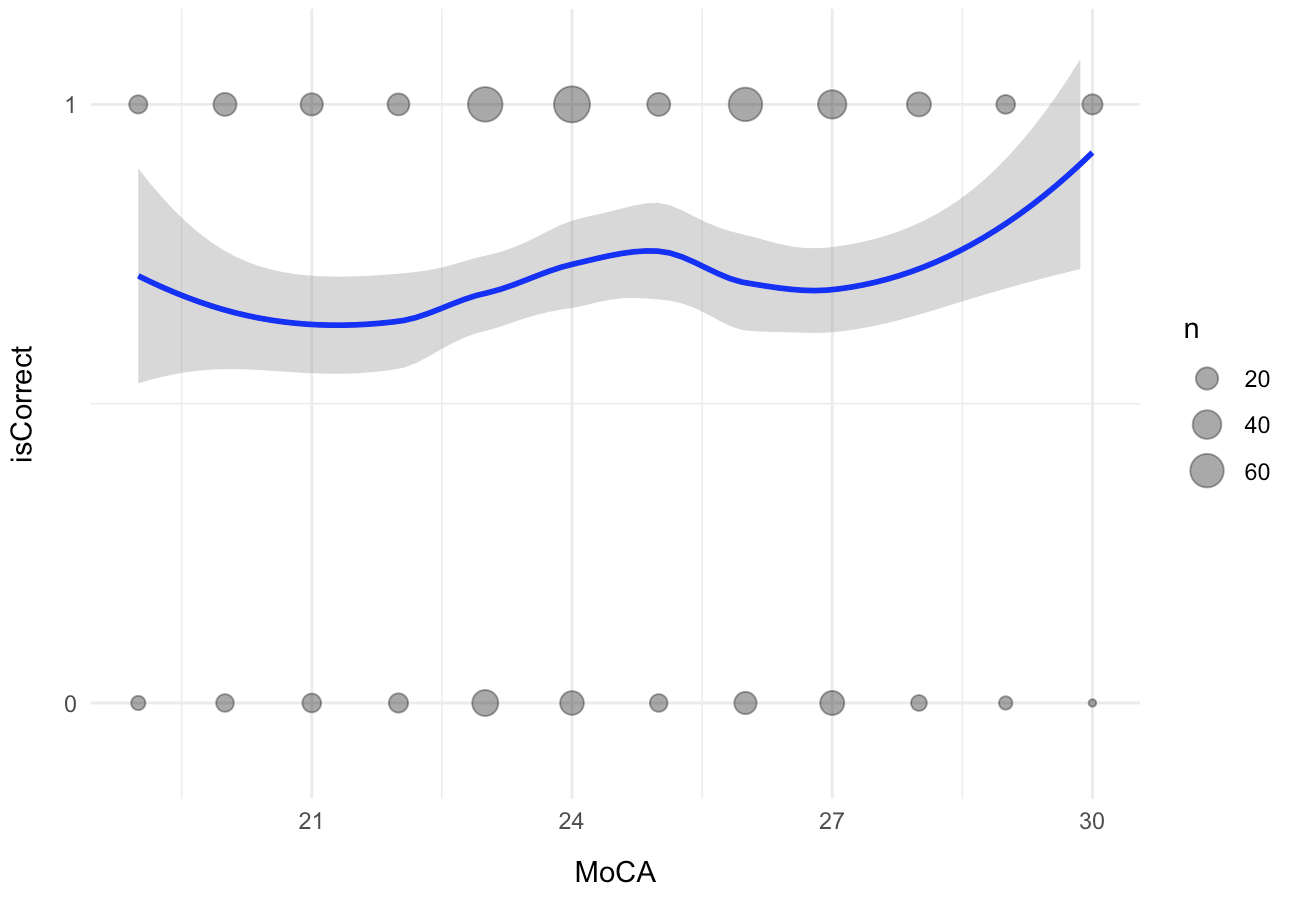


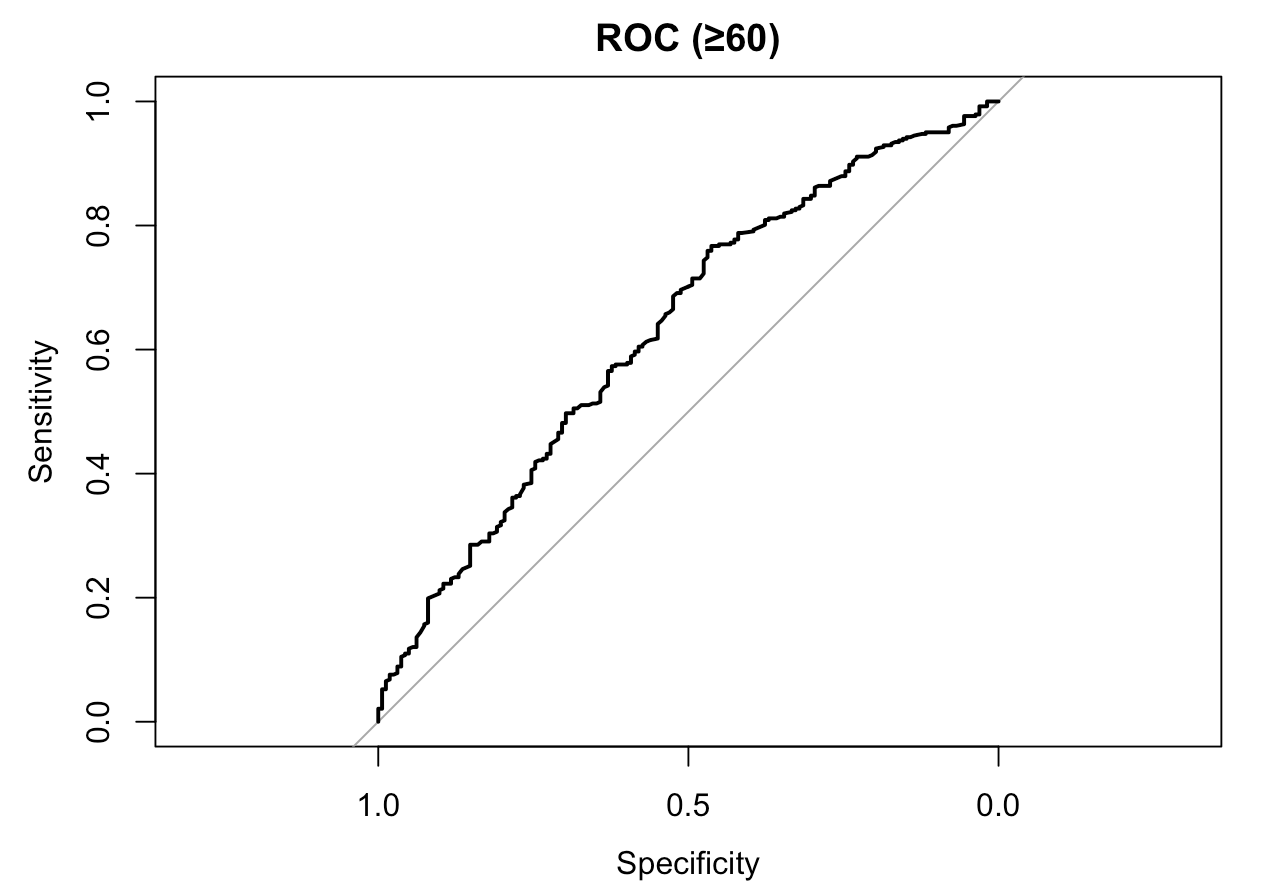

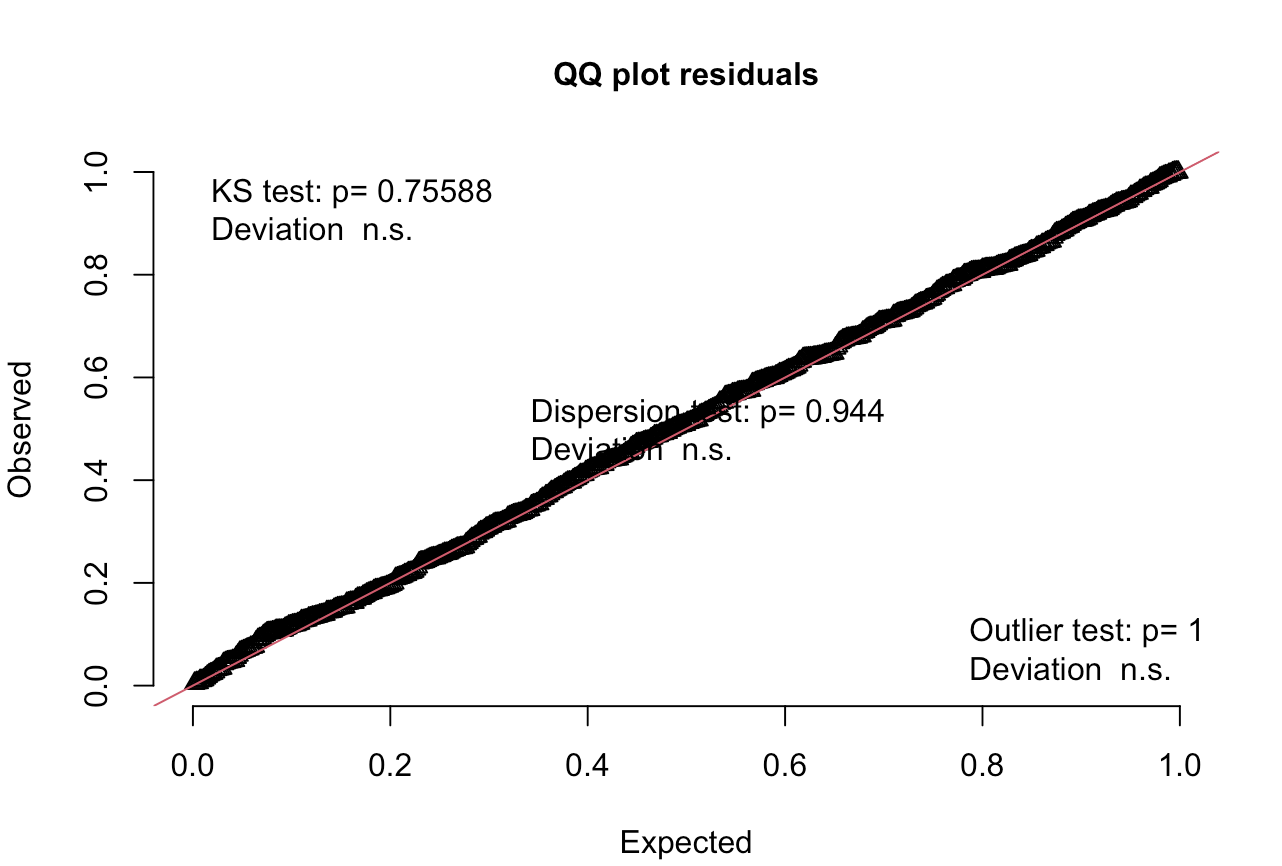


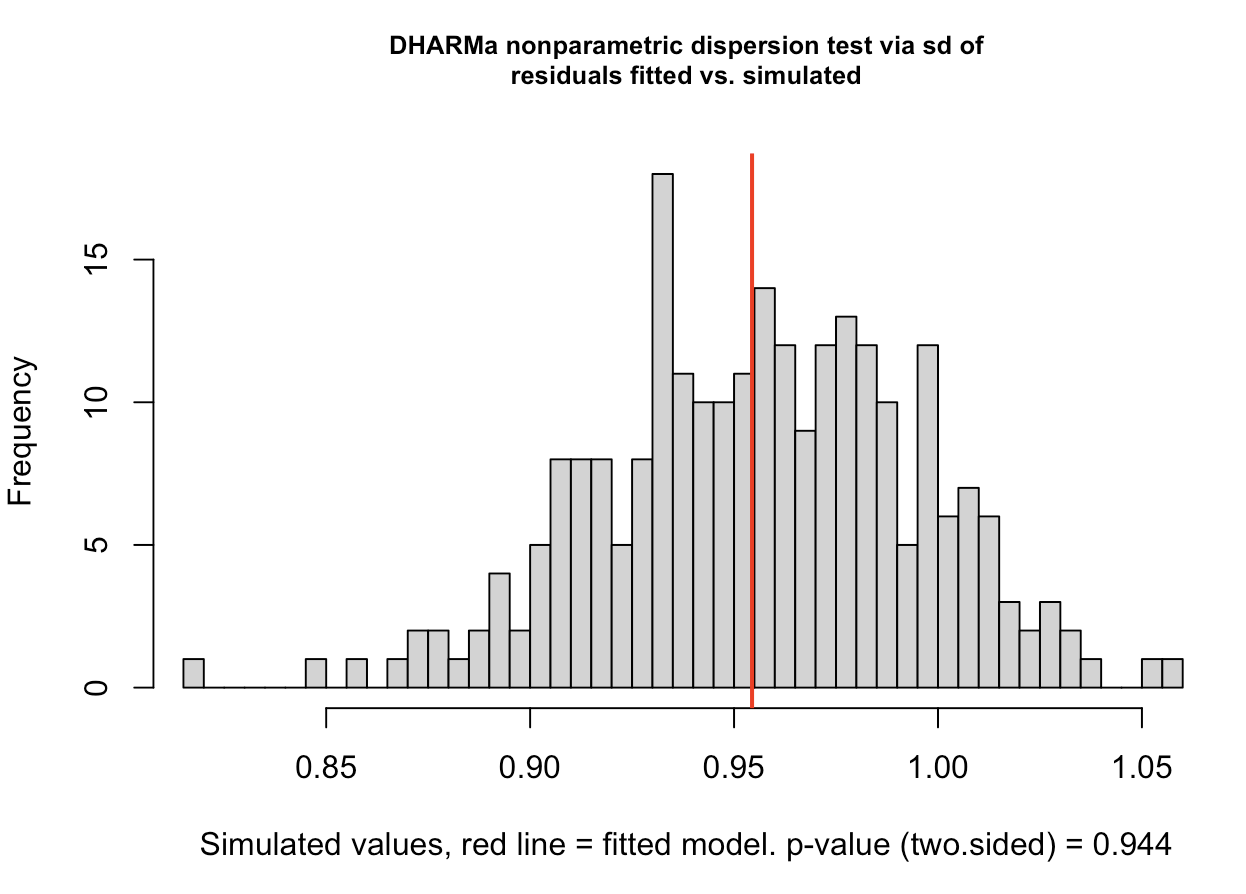

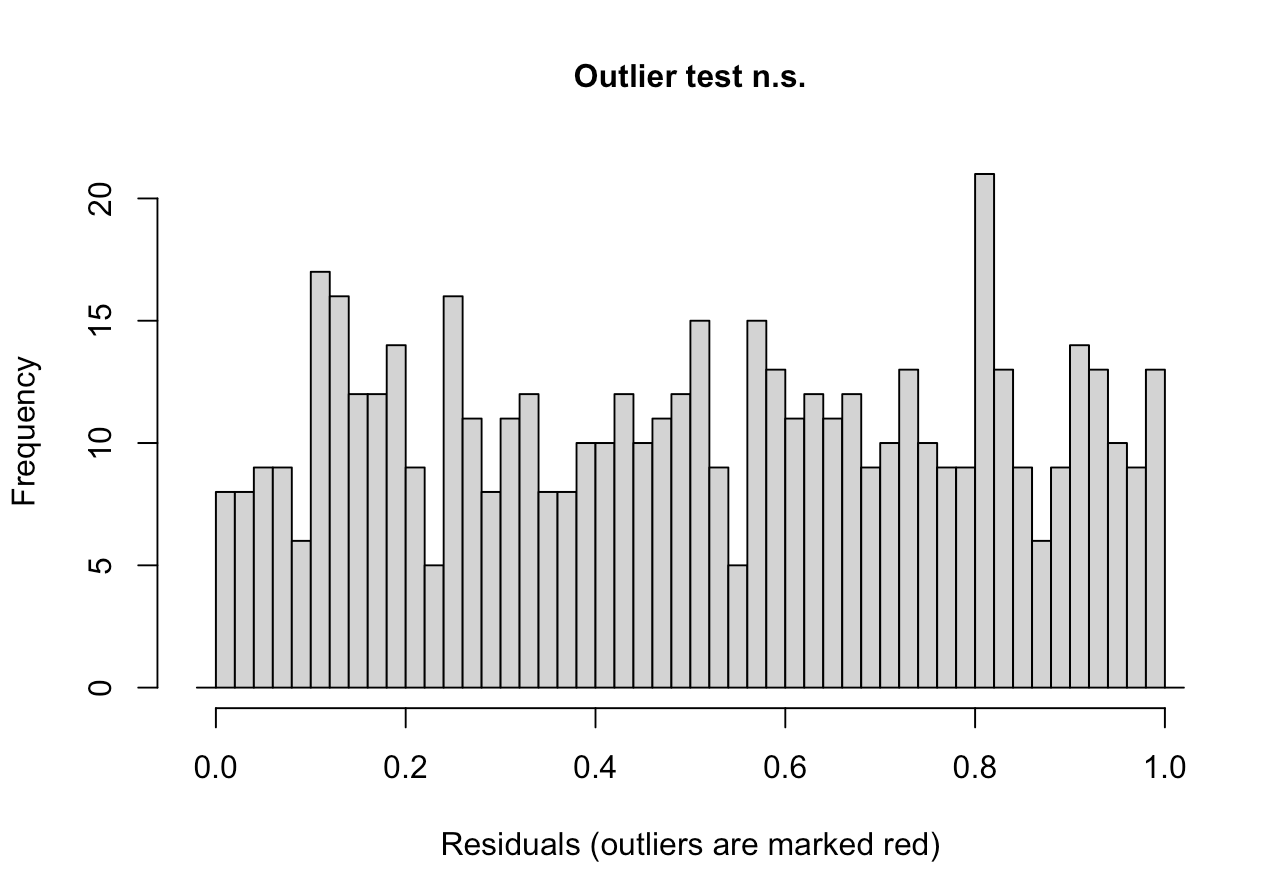


**Supplemental Figure 7. Overview of model predictions and diagnostics for participants ≥ 60 years.** Top left: relationship between correct responses (isCorrect) and ΔECIE2000. Top right: relationship between correct responses and MoCA scores. Middle left: ROC curve assessing model discrimination. Middle right: DHARMa residual diagnostics checking for deviations from uniformity. Bottom left: DHARMa dispersion test assessing over- or under-dispersion. Bottom right: DHARMa outlier test identifying influential observations.

**References**

1. Arnhardt S, Fu LW, Kornhuber J, et al. Sniffing colors—color associations for descriptors and odors of the MONEX-40 test. *Chem Senses* 2025; 50: bjaf004.

2. Croy I and Hummel T. Olfaction as a marker for depression. *J Neurol* 2017; 264: 631–638.

3. Beck A, Steer R and Brown G. *BDI-II, Beck depression inventory manual 2nd ed.* San Antonio, TX, Boston, MA: Psychological Corporation, Harcourt Brace, 1996.

4. Beck AT. An Inventory for Measuring Depression. *Arch Gen Psychiatry* 1961; 4: 561.

5. Hummel T, Rosenheim K, Konnerth C-G, et al. Screening of olfactory function with a four-minute odor identification test: reliability, normative data, and investigations in patients with olfactory loss. *Ann Otol Rhinol Laryngol* 2001; 110: 976–981.

6. Hummel T, Sekinger B, Wolf SR, et al. ‘Sniffin’ Sticks’: olfactory performance assessed by the combined testing of odour identification, odor discrimination and olfactory threshold. *Chem Senses* 1997; 22: 39–52.

7. Nasreddine ZS, Phillips NA, Bédirian V, et al. The Montreal Cognitive Assessment, MoCA: a brief screening tool for mild cognitive impairment. *J Am Geriatr Soc* 2005; 53: 695–699.

8. Kuchenbecker J and Broschmann D. *Tafeln zur Prüfung des Farbensinnes / Farbensehens*. Stuttgart: Thieme, 2016.

9. Kalmus H. The familial distribution of congenital tritanopia. *Ann Hum Genet* 1955; 20: 39–56.

10. Apri M, Lousmono AL, Suandi D, et al. Understanding the spread of color blindness via population genetics model. *Commun Math Biol Neurosci* 2024; 2024: 28.

11. Sohrabi HR, Gavett BE, Weinborn M, et al. The McCusker Subjective Cognitive Impairment Inventory (McSCI): a novel measure of perceived cognitive decline. *Age Ageing* 2024; 53: afae138.
